# Determining breast cancer biomarker status and associated morphological features using deep learning

**DOI:** 10.1038/s43856-021-00013-3

**Published:** 2021-07-14

**Authors:** Paul Gamble, Ronnachai Jaroensri, Hongwu Wang, Fraser Tan, Melissa Moran, Trissia Brown, Isabelle Flament-Auvigne, Emad A. Rakha, Michael Toss, David J. Dabbs, Peter Regitnig, Niels Olson, James H. Wren, Carrie Robinson, Greg S. Corrado, Lily H. Peng, Yun Liu, Craig H. Mermel, David F. Steiner, Po-Hsuan Cameron Chen

**Affiliations:** 1 Google Health, Palo Alto, CA USA; 2 Google Health via Vituity, Emeryville, CA USA; 3 Department of Pathology, School of Medicine, University of Nottingham, Nottingham, UK; 4 John A. Burns University of Hawaii Cancer Center, Honolulu, HI USA; 5 Department of Pathology, Magee‐Womens Hospital of UPMC, Pittsburgh, PA USA; 6 Diagnostic and Research Institute of Pathology, Medical University of Graz, Graz, Austria; 7 Defense Innovation Unit, Mountain View, CA USA; 8 Henry M. Jackson Foundation, Bethesda, MD USA; 9 Laboratory Department, Naval Medical Center San Diego, San Diego, CA USA

**Keywords:** Breast cancer, Pathology

## Abstract

**Background:**

Breast cancer management depends on biomarkers including estrogen receptor, progesterone receptor, and human epidermal growth factor receptor 2 (ER/PR/HER2). Though existing scoring systems are widely used and well-validated, they can involve costly preparation and variable interpretation. Additionally, discordances between histology and expected biomarker findings can prompt repeat testing to address biological, interpretative, or technical reasons for unexpected results.

**Methods:**

We developed three independent deep learning systems (DLS) to directly predict ER/PR/HER2 status for both focal tissue regions (patches) and slides using hematoxylin-and-eosin-stained (H&E) images as input. Models were trained and evaluated using pathologist annotated slides from three data sources. Areas under the receiver operator characteristic curve (AUCs) were calculated for test sets at both a patch-level (>135 million patches, 181 slides) and slide-level (*n* = 3274 slides, 1249 cases, 37 sites). Interpretability analyses were performed using Testing with Concept Activation Vectors (TCAV), saliency analysis, and pathologist review of clustered patches.

**Results:**

The patch-level AUCs are 0.939 (95%CI 0.936–0.941), 0.938 (0.936–0.940), and 0.808 (0.802–0.813) for ER/PR/HER2, respectively. At the slide level, AUCs are 0.86 (95%CI 0.84–0.87), 0.75 (0.73–0.77), and 0.60 (0.56–0.64) for ER/PR/HER2, respectively. Interpretability analyses show known biomarker-histomorphology associations including associations of low-grade and lobular histology with ER/PR positivity, and increased inflammatory infiltrates with triple-negative staining.

**Conclusions:**

This study presents rapid breast cancer biomarker estimation from routine H&E slides and builds on prior advances by prioritizing interpretability of computationally learned features in the context of existing pathological knowledge.

## Plain language summary

Breast cancer diagnosis and characterization involves evaluation of marker proteins found inside or on the surface of tumor cells. Three of the most important markers are estrogen receptor (ER), progesterone receptor (PR) and a receptor called HER2. The levels of these markers can influence how a person with breast cancer is treated in the clinic. This study explored the ability of machine learning – whereby computer software is trained to recognise and classify particular image features - to determine the status of these markers in digitized images, without the need for tissue stains. Our results demonstrate that machine learning can automatically predict the status of ER, PR and HER2 in pathology images and further testing identifies specific image features which enable these predictions. This type of approach may decrease costs and timelines and enable improved quality control in marker detection.

## Abstract (web summary)

Gamble and Jaroensri et al. develop deep learning systems to predict breast cancer biomarker status using H&E images. Their models enable slide-level and patch-level predictions for ER, PR and HER2, with interpretability analyses highlighting specific histological features associated with these markers.

## Introduction

Clinical biomarkers are critically important in the diagnostic workup and treatment of breast cancer. In breast cancer, three molecular biomarkers form a cornerstone for optimized clinical decision making by providing prognostic information and predicting response to specific therapies. These biomarkers include estrogen receptor (ER), progesterone receptor (PR), and human epidermal growth factor receptor 2 (HER2)^1^. In current clinical practice, biomarker status is typically determined by histological inspection of immunohistochemistry (IHC) stained tissue using separate IHC stains for each biomarker.

Biomarker profiles are known to be correlated with histologic features in breast cancer^2–5^. This is further recognized in the 2019 American Society of Clinical Oncology (ASCO) and College of American Pathologists (CAP) guidelines, which recommend follow up for observed discordance between ER status and histologic findings, such as low grade but ER-negative carcinoma^6^. Such follow up includes a second review or repeat IHC staining and is meant to help ensure that technical issues, tumor heterogeneity, or interpretation variability are ruled out before the biomarker status is used for treatment decisions.

With the adoption of digital workflows in histopathology and recent advancements in machine learning, initial efforts have explored the possibility of using algorithms to predict biomarker status from hematoxylin and eosin (H&E)-stained tissue in breast cancer^7–9^ and other cancer types^10,11^. Not only could this approach provide a more efficient and accessible option than IHC, but it also provides the intriguing scientific potential to identify morphological features that correlate with biomarker status. Explainability efforts have provided some insights into the features learned via slide-level determination of biomarker status^12^, but further investigation of localized feature-prediction associations as well as comparison of features learned by different modeling approaches remains an important next step. Such work may also help pathologists identify morphological findings that further inform histology-biomarker discordances and reduce incorrect biomarker status reporting.

In this study, we develop models to predict three clinically relevant breast cancer biomarkers from H&E images, providing biomarker predictions for localized tumor regions as well as the slide-level summarization that has been the focus of prior studies. We also leverage multiple model-interpretation techniques to further investigate the associations between morphologic features and biomarker status predictions learned by the models. Taken together, our modeling and interpretability results provide both qualitative and quantitative assessment of morphological features relevant to biomarker prediction in breast cancer, highlighting that deep learning approaches in pathology can be accurate, informative, and interpretable.

## Methods

### Datasets

De-identified breast cancer data for this study came from three sources (Table 1): a tertiary teaching hospital, a medical laboratory, and TCGA^13, 14^. The teaching hospital contributed both formalin-fixed paraffin-embedded (FFPE) tissue blocks (from which new IHC-stained slides could be prepared) and archived H&E-stained slides. The medical laboratory contributed only tissue blocks and TCGA represents only archival H&E slides. Pathology reports were available for cases from all three sources. Inclusion criteria for the H&E images required the presence of invasive carcinoma in primary breast tissue specimens, as determined by pathologist review.

**Table 1:** Dataset summary for slides and cases used in model development and evaluation.

		Tertiary teaching hospital	Medical laboratory	Tertiary teaching hospital	Tertiary teaching hospital	TCGA(36 sites)
DLS Stage 1 (patch-level): uses paired H&E and IHC slides from custom sectioning protocol				NA		
	No. of cases(train / tune / test)	70 / 30/ 64	70 / 30/ 0			
	No. of H&E slides(Train / tune / test)	205 / 80/ 181	206 / 85/ 0			
	No. of patches	See Table 2				
DLS Stage 2 (slide-level): uses biomarker status from the original report		Train		Tune	Test	
	No. of cases	164	100	164**	340	909
	No. of H&E slides	466	291	1,377	2,313	961
	ER status* (pos / neg)	103 / 47	91 / 7	103 / 47	280 / 58	679 / 191
	PR status* (pos / neg)	93 / 56	85 / 13	93 / 56	251 / 87	573 / 283
	Her2 status* (pos / neg)	16 / 34	4 / 81	16 / 34	11 / 78	131 / 739
	Nottingham grade 1 / 2 / 3	46 / 69/ 49	44 / 36/ 20	46 / 69/ 49	135 / 126/ 79	387 / 295 / 227

^*^The total case counts for each biomarker are different based on availability of biomarker status in original pathology reports.

**Stage 2 tune set includes the same cases as stage 2 train set from Tertiary Teaching Hospital dataset but different tumor-containing slides from those cases.

Slides from the teaching hospital and medical laboratory (whether archival or newly prepared) were scanned by Aperio AT2 digital scanners, and TCGA whole-slide images were digitized by Aperio and 3DHistech scanners and obtained via the Genomic Data Commons Data Portal (https://gdc.cancer.gov). The study protocol was approved and informed consent was waived by the Naval Medical Center San Diego (NMCSD) Institutional Review Board (IRB). This IRB approval covered the use of de-identified cases for the data from the tertiary hospital, the medical laboratory, and TCGA as used in this study.

**Fig. 1:**
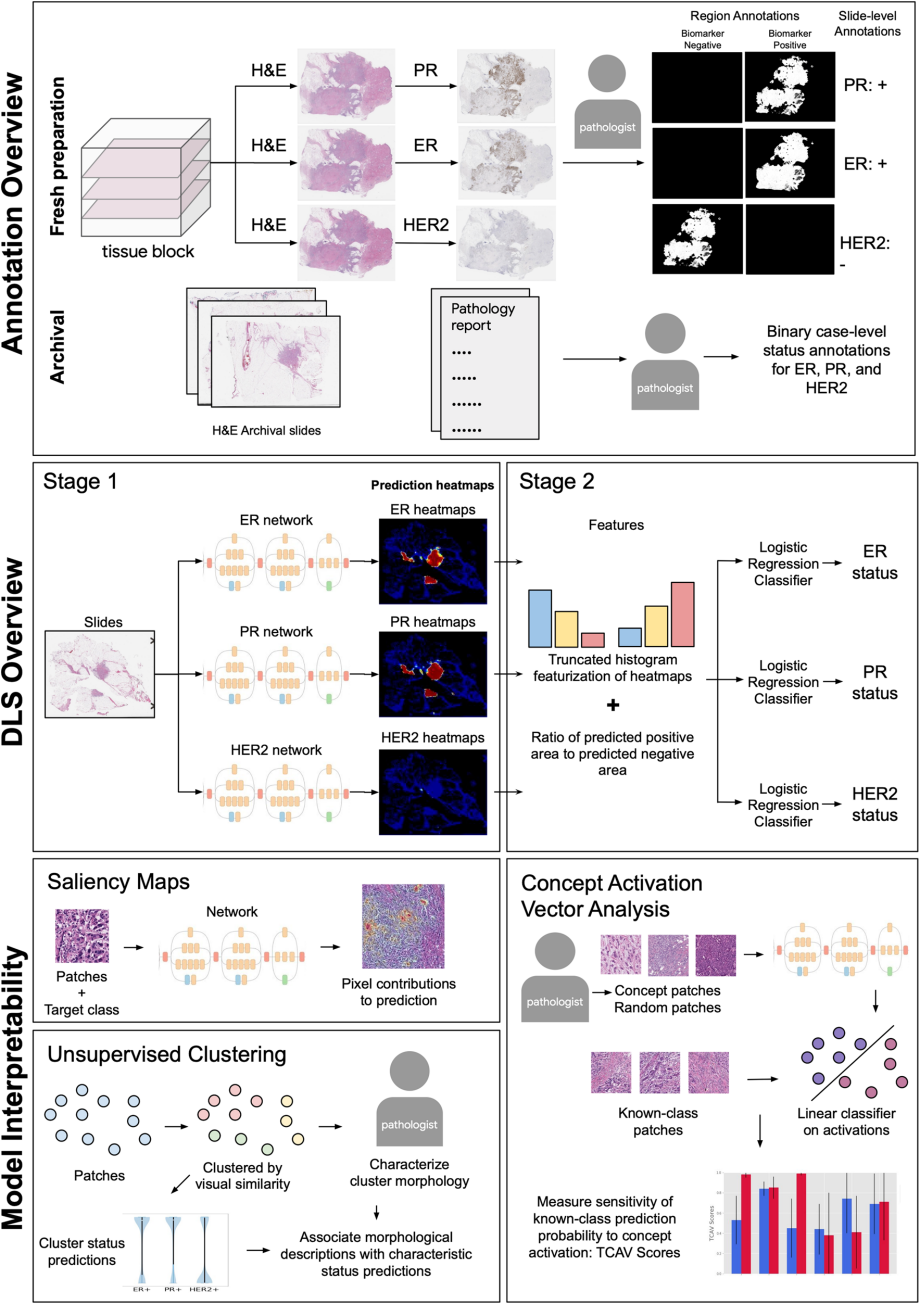
Overview of data annotation, the two-stage deep learning system (DLS), and interpretability techniques. Annotation overview: paired H&E and IHC images were used to develop regional biomarker annotations (see Supplementary Fig. 4). Case-level biomarker status labels were obtained from available pathology reports. DLS overview: a model based on the Inception-v3 architecture was developed for each biomarker. Model interpretability: saliency maps and unsupervised clustering provided an exploratory approach to interpretability, while concept activation vector analysis provided hypothesis-driven analysis of features associated with biomarker predictions. H&E Hematoxylin and Eosin, ER Estrogen Receptor, PR Progesterone Receptor, HER2 human epidermal growth factor receptor 2.

First, to develop the patch-level DLS that predicts the biomarker status of each region of tissue, we prepared new, paired H&E and IHC slides from available tissue blocks from the hospital and laboratory. For each block, three 4 μm serial sections were prepared (Fig. 1) and each slide was first stained with H&E, digitized, then de-stained and stained with IHC for ER, PR, and HER2, respectively. Quality review of these IHC-stained slides was performed by multiple pathologists for a limited number of sample specimens prior to adopting this approach and showed comparable results to serial section staining. Quality assurance requirements were also employed throughout, including recutting and re-staining for a small number of sections with poor staining of the initial H&E or re-stained IHC. After digitization and alignment (see Supplementary Methods), these adjacent sections enabled simultaneous review of H&E and corresponding IHC for precise determination and manual annotation of biomarker status. The patch-level stage of the DLS was trained using pathologist-labeled patches from 140 blocks, tuned using 60 blocks, and tested on 64 blocks (see Table 2 for numbers of labeled patches).

**Table 2:** Data summary for patches used in model development and evaluation.

Biomarker label	Train	Tune	Test
ER positive / negative / other	71.2 M / 25.5 M / 204.8 M	31.7 M / 3.6 M / 71.2 M	24.5 M / 18.3 M / 101.8 M
PR positive / negative / other	79.5 M / 16.1 M / 198.2 M	31.5 M / 2.6 M / 72.4 M	35.0 M / 7.9 M / 93.0 M
HER2 positive / negative / other	34.7 M / 64.3 M / 196.8 M	10.5 M / 28.6 M / 66.5 M	15.0 M / 29.4 M / 99.1 M

The second stage of the DLS, that provides slide-level summarization of biomarker status, was trained on biomarker status extracted from the pathology reports. This slide-level stage of the DLS was trained on 757 slides, tuned on 1377 slides, and tested on 3274 slides (see Tables 1 and 2 for breakdown by source).

### Biomarker labels

Labels were provided by a cohort of 16 pathologists (median years of post training experience 8.5 years, range 3–21). For patch-level labels, pathologists were provided pairs of aligned H&E and IHC images to enable easy review of IHC status for specific regions of each H&E slide. Contiguous regions of invasive carcinoma were identified, without enrichment or selection of specific subtypes, and annotated as positive or negative for biomarker status using a 1% threshold for ER and PR, and a 10% threshold of complete circumferential staining for HER2. For the slide-level review of biomarker status, pathologists reviewed the IHC slides when available and assigned positive, negative, or indeterminate for ER and PR and 0, 1+, 2+, or 3+ for HER2 as per CAP guidelines^15^.

For all archival cases, pathologists also reviewed de-identified pathology reports and extracted the reported biomarker status. For ER and PR, each case was categorized as either positive, negative, or unavailable. For HER2 status, IHC results were reported as positive, negative, equivocal, or unavailable. For equivocal cases, HER2 fluorescence in situ hybridization (FISH) results from pathology reports were also recorded as positive, negative, or unavailable and used when available.

### Deep Learning System (DLS) development

We developed a separate DLS for each biomarker (ER, PR, and HER2) to enable exploration of the possibility that different morphological features might be associated with each biomarker. Each DLS consists of two stages: the first is a deep convolutional neural network based on the Inception-V3^16^ architecture, that operates on image patches cropped from the whole slide image. Each input image patch was of size 512 × 512 pixels at 5X magnification (1024 µm wide at 2 µm/pixel). The patches used for model development were randomly sampled across the complete training set without any enrichment for morphological features or histologic subtypes. The model was trained to categorize each patch as one of three categories: biomarker positive invasive carcinoma, biomarker negative invasive carcinoma, and “non-tumor” (i.e., not invasive carcinoma). After the softmax layer, all predictions were in the range [0,1].

Training labels were collected as described above for each biomarker (one H&E-stained section labeled for each of the three biomarkers). To increase the number of labeled patches, pathologist-annotated H&E regions for each biomarker were replicated to the two available serial H&E images as a form of data augmentation that was found empirically to improve performance on a tuning set. For example, if a region of the H&E section was labeled as ER-positive (based on the paired IHC image), that ER-positive region label was propagated to the two serial H&E sections (even though those specific sections were not stained or evaluated for ER status). Further training details and other hyperparameters are provided in Supplementary Table 1.

The second stage utilizes features extracted from the patch-level output (i.e., heatmaps) to classify each slide as positive or negative for each biomarker status. For each biomarker, three sets of features were used: a single feature indicating the ratio of biomarker-positive vs biomarker-negative areas, and two normalized histograms of patch-level predicted values: one for biomarker positivity and one for biomarker negativity.

To compute biomarker-positive/negative area from continuous stage-1 predictions, we defined a single threshold *t* for both biomarker-positive (or negative) patches. For example, patches with predicted probability of ER-positivity >= *t* were considered ER-positive, and patches with predicted probability of ER-negativity >= *t* were considered ER-negative. Patches not meeting either threshold were considered “non-tumor” (i.e., not invasive carcinoma). The ratio of positive versus negative patches was used as the first feature for the stage 2 model. Next, we incorporated information about the full spectrum of predictions so that “borderline” predictions were not discarded and to reduce dependency on the exact threshold *t*. Specifically, we considered *b* evenly spaced histograms of patch-level predicted probabilities for both biomarker-positivity and biomarker-negativity. Prediction outputs below 0.1 were discarded because they tended to indicate non-invasive carcinoma (i.e., neither biomarker positive nor negative). The histogram values were then normalized by the sum of biomarker-positive and biomarker-negative patches (based on the above threshold *t*). Finally, these features were used as input to a regularized logistic regression model (implemented in Python’s sklearn library, v0.23.2).

The threshold *t*, number of histogram bins *b*, L1 vs L2 regularization, and regularization strength *C* for the logistic regression were all tuned using 10-fold cross validation on the slide-level train and tune dataset (Table 1). The final hyperparameters used were: *t* = 0.7, *b* = 5 for ER and PR, *b* = 7 for HER2, L1 regularization with *C* = 0.077, 0.045, and 0.024 for ER, PR, and HER2, respectively.

### Model interpretability

#### Testing association of DLS patch-level predictions with specific histologic concepts

To evaluate the association of specific histomorphological features with the biomarker status predictions made by our models, we performed analysis of concept activation vectors (CAV analysis)^17^. Briefly, CAV quantitatively evaluates the degree to which a DLS associates a user-specified ‘concept’ with a particular predicted classification. This approach is hypothesis driven and thus requires a proposed set of concepts to test. Based on discussions with experienced breast subspecialist pathologists regarding known or likely associations with biomarker status, we identified six concepts for CAV analysis: high-grade carcinoma, low-grade carcinoma, invasive lobular carcinoma, DCIS, tumor-adjacent desmoplastic stromal changes, and TILs.

CAV analysis requires three categories of patches: patches representing particular histomorphological concepts (concept patches), patches known to be specific biomarker classes (known-class patches), and patches selected randomly from the entire dataset (random patches) to serve as a control. A minimum of 100 concept patches were randomly sampled from pathologist-annotated regions for the six concepts across 45 slides from the slide-level tuning set. Known-class patches consisted of 500 patches randomly sampled from the annotated positive and negative regions for ER, PR, and HER2 in the slide-level tuning set. Random patches consisted of 10 sets of 500 patches each, sampled randomly from all tissue areas.

To conduct CAV analysis, we generate activations at the final concatenation layer between the convolutional blocks and the fully connected layers for all concept patches and random patches, train a set of 20 linear support vector machine classifiers to distinguish between a sampling of random and concept activations (see Supplementary Methods for more details), and then measure the directional derivative of the model’s prediction for a given class along a vector orthogonal to the SVM decision boundaries (the Concept Activation Vector)^13^. For each concept–biomarker pair, we report the TCAV score, which is defined as the fraction of patches of a known class that have a positive derivative.

#### Unsupervised clustering

In this analysis, all patches from the patch-level training and tuning sets that were labeled as invasive carcinoma were clustered using a deep-learning based model that has been previously shown to be able to retrieve visually-similar histopathology images patches^18, 19^. This model uses as input patches of size 299 × 299 pixels; therefore to obtain embeddings for our model’s input patches (512 × 512 pixels), we concatenated the image-similarity model’s embeddings for the 2 × 2 overlapping patches that constitute each of our patches.

We qualitatively selected the minimal number of clusters (25) that maintained within-cluster visual consistency. Each cluster contained 900–3000 patches, representing 30–100 cases. Next, 5 pathologists, blinded to the DLS predictions, reviewed 10 patches from each cluster. These patches were selected to be those closest to the cluster center while maintaining that each patch was from a distinct case. The pathologists provided both free-text characterization of morphological features and filled out a structured survey of histologic features.

To better understand which clusters were most similar in terms of predicted biomarker statuses, we next computed the patch-level predictions for ER, PR, and HER2 biomarker-positivity for every patch from the tune set, obtaining a distribution of biomarker status predictions for each cluster. We then used hierarchical clustering based on the average linkage to group these clusters. The distance metric used was the sum of the earth mover’s distance across all three biomarker-positivity predictions. Finally, to define each group resulting from the hierarchical clustering, we manually examined the mean and median biomarker-positivity scores of each group.

#### Saliency maps

Finally, to better understand the predictions at the pixel level, we leveraged SmoothGrad^20^. Briefly, SmoothGrad calculates the gradient of output prediction with respect to the input pixels, and averages these gradients across multiple copies of the input image (*n* = 8 in our work), each with pixel-wise Gaussian noise added. Saliency maps were generated for a minimum of 100 patches per biomarker, 50 for each of positive and negative, a subset was manually chosen for independent review by two pathologists. The pathologists were presented with a high magnification version of the model input patch and the corresponding saliency map overlay, and asked to qualitatively assess the most salient regions on the patch.

### Statistical analysis

Model performance was evaluated for both patch-level and slide-level predictions by calculating AUC for each biomarker. Confidence intervals for patch-level and slide-level AUCs were computed via bootstrapping by sampling with replacement (1000 iterations) using Python’s sklearn package, v0.23.2. Reported patch level AUCs are one-vs-all for the biomarker positive invasive carcinoma class. Slide-level AUCs represent binary classification of biomarker positive versus negative. Confidence intervals for the TCAV analyses were computed using the same method with 100 iterations.

## Results

Our approach involves a 2-stage deep learning system (DLS) for each biomarker. The first stage predicts the local biomarker status for individual, cropped image patches representing small regions of tissue. The output of this prediction is one of three classes: biomarker positive tumor, biomarker negative tumor, or non-tumor. The second stage of the DLS predicts the slide-level biomarker status using the predictions of the first stage across every patch in the slide (Fig. 1, Methods).

### Patch-level model status prediction

**Fig. 2:**
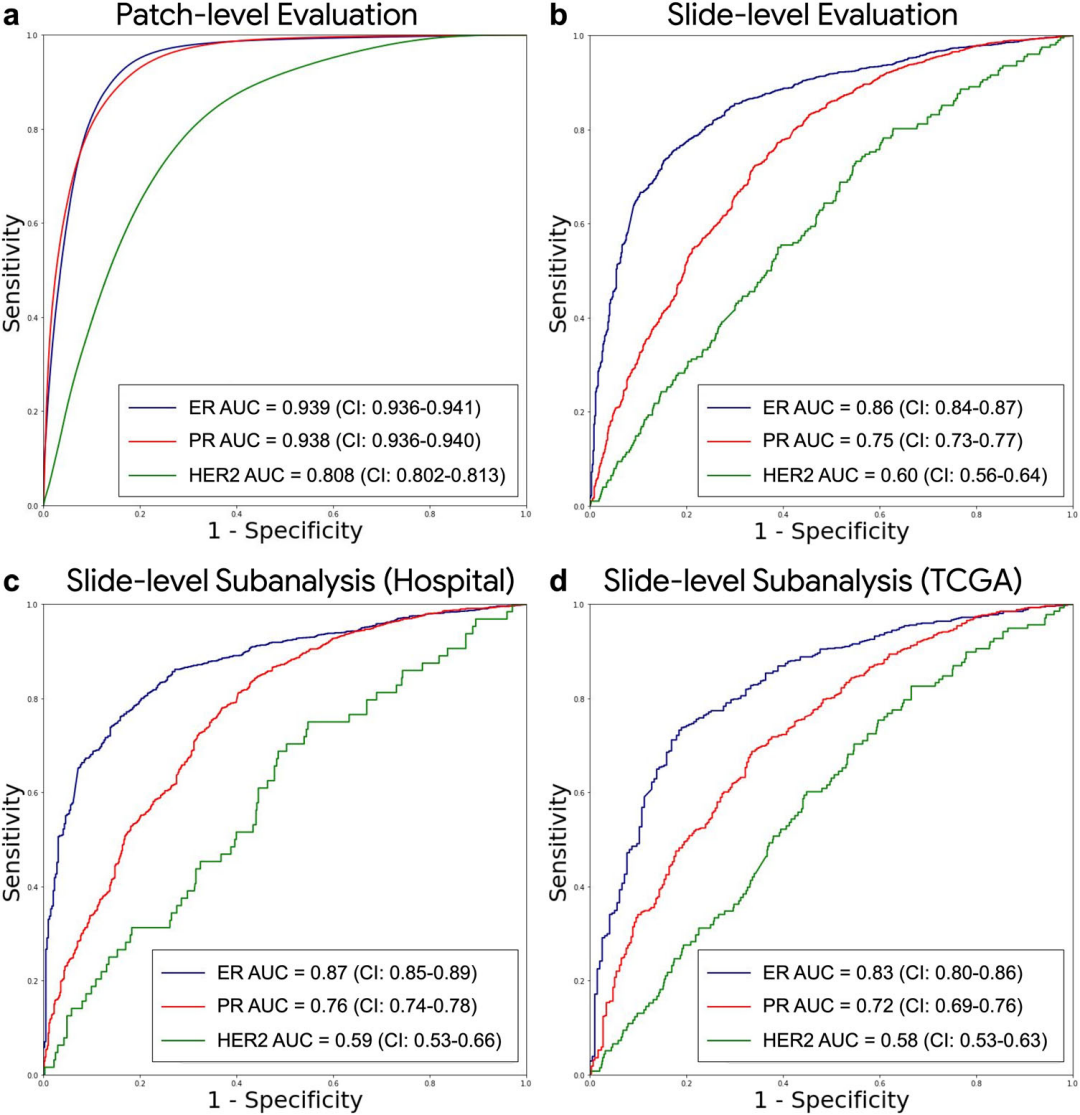
Deep learning system performance. ROC curves for model performance are shown for **a** patch-level predictions across all tissue patches of WSIs, **b** slide-level predictions of the stage 2 model output on the full test set, **c**–**d** subanalysis for slide-level performance on the independent data sources of the slide level test set. Patch-level analysis represents 3-class performance (biomarker positive invasive carcinoma, biomarker negative invasive carcinoma, or non-tumor) and slide-level performance represents positive versus negative classification for biomarker status (all slides in the final datasets contain tumor). Binary patch-level performance for biomarker status across tumor regions only are shown in Supplementary Fig. 5. The number of slides, cases, and patches used for this analysis are indicated in Tables 1 and 2. ER Estrogen Receptor, PR Progesterone Receptor, HER2 human epidermal growth factor receptor 2.

**Fig. 3:**
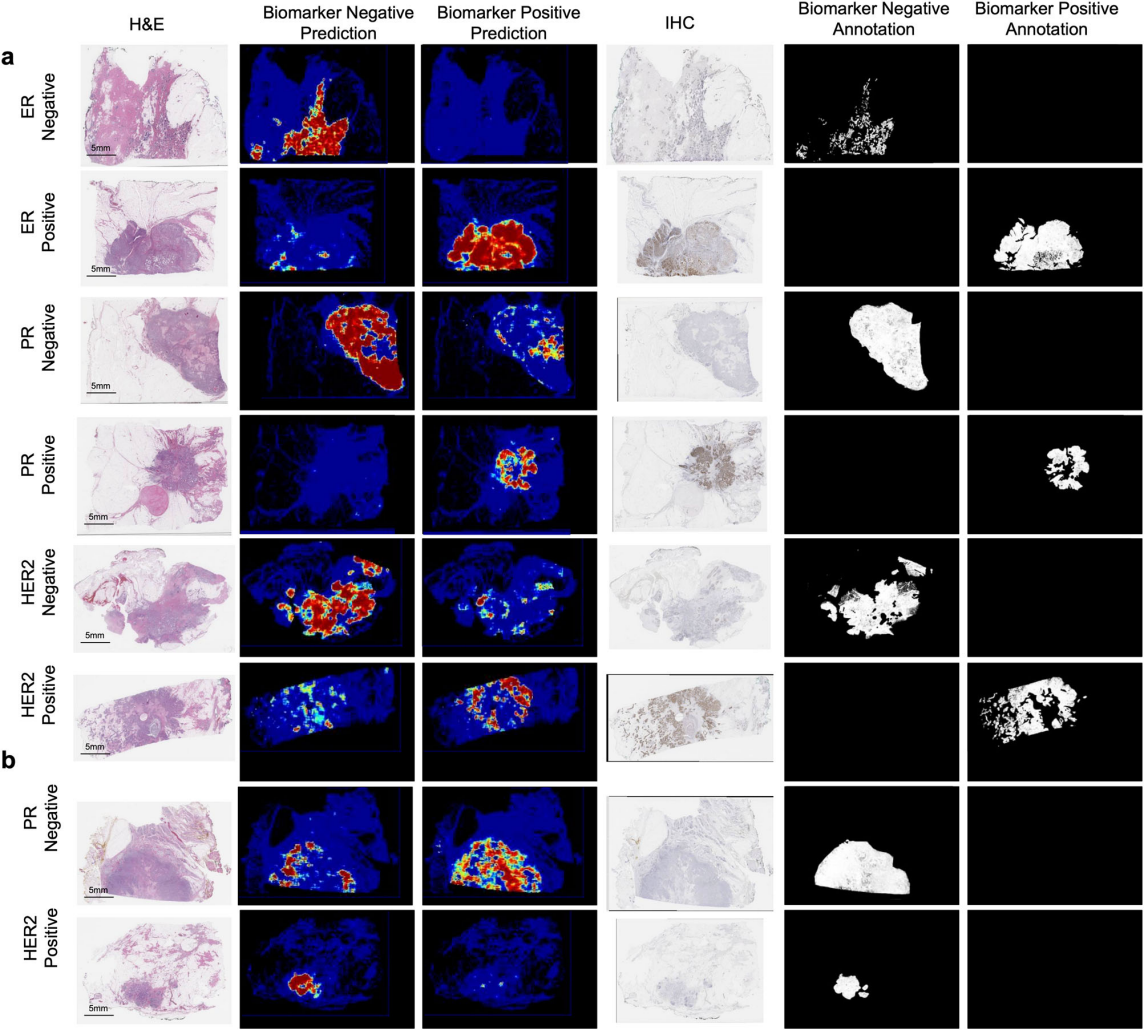
Visualization of predictions and annotations. **a** Sample cases for which DLS predictions are concordant with region-level pathologist annotations. **b** Sample cases for which DLS predictions are discordant with region-level pathologist annotations. Predictions are represented by heatmaps with colors ranging from blue (low predicted probability) to red (high predicted probability), and with black indicating non-tissue. In the annotation masks (black and white), annotations are represented by white regions, and corresponding labels indicated in the column header. H&E Hematoxylin and Eosin, IHC Immunohistochemistry, DLS Deep Learning System, ER Estrogen Receptor, PR Progesterone Receptor, HER2 human epidermal growth factor receptor 2.

The first stage of the DLS was developed (trained and tuned) using 1.21 billion patches from 576 slides across 200 cases, and evaluated on a test set of all patches from 181 slides across 64 cases (Table 1 and Supplementary Table 2). We next report one versus all classification performance across all patches (biomarker positive tumor, biomarker negative tumor, or non-tumor). The patch-level area under the receiver operating characteristic curves (AUCs) were 0.939 (95%CI 0.936–0.941), 0.938 (95%CI 0.936–0.940), and 0.808 (95%CI 0.802–0.813) for ER, PR, and HER2, respectively (Fig. 2a). Examples of the patch-level predictions along with the corresponding IHC images are shown in Fig. 3 and Supplementary Figs. 1 and 2. We observed that heterogeneous staining was indeed associated with heterogeneous predictions, and this was in contrast to the uniformly positive patch-level predictions observed for the homogeneous cases (Supplementary Figs. 1 and 2).

### Slide-level biomarker status prediction

The second stage of the DLS, which provides slide-level biomarker status predictions, was developed (trained and tuned) using 2134 slides from 264 cases, and evaluated on a test set containing a total of 3274 slides from 1249 cases across a tertiary hospital and The Cancer Genome Atlas (TCGA^13, 14^, representing 36 unique tissue source sites), summarized in Table 1. On the combined test set, the AUCs for binary biomarker status classification were 0.86 (95% CI 0.84–0.87), 0.75 (95% CI 0.73–0.77), and 0.60 (95% CI 0.56–0.64) for ER, PR, and HER2, respectively (Fig. 2b).

We also evaluated AUC separately for the two test set data sources, observing AUCs of 0.87 (95% CI 0.85–0.89) for ER, 0.76 (95% CI 0.74–0.78) for PR, and 0.59 (95% CI 0.53–0.66) for HER2 for the tertiary hospital (Fig. 2c), and 0.83 (95% CI 0.80–0.86) for ER, 0.72 (95% CI 0.69–0.76) for PR, and 0.58 (95% CI 0.53–0.63) for HER2 for TCGA (Fig. 2d).

### Model interpretability

To further understand the biomarker predictions, we leveraged three approaches for model interpretability. First, we quantitatively tested whether the features used by the trained model corresponded to existing histologic concepts using an approach called TCAV^17^ (Testing with Concept Activation Vectors). Second, we performed histopathologic characterization of features shared by patches clustered by visual similarity and grouped by predicted biomarker patterns. Finally, we explored what features in image patches most strongly impacted biomarker predictions using a pixel-based saliency approach (SmoothGrad^20^).

#### Testing association of DLS patch-level predictions with specific histologic concepts

**Fig. 4:**
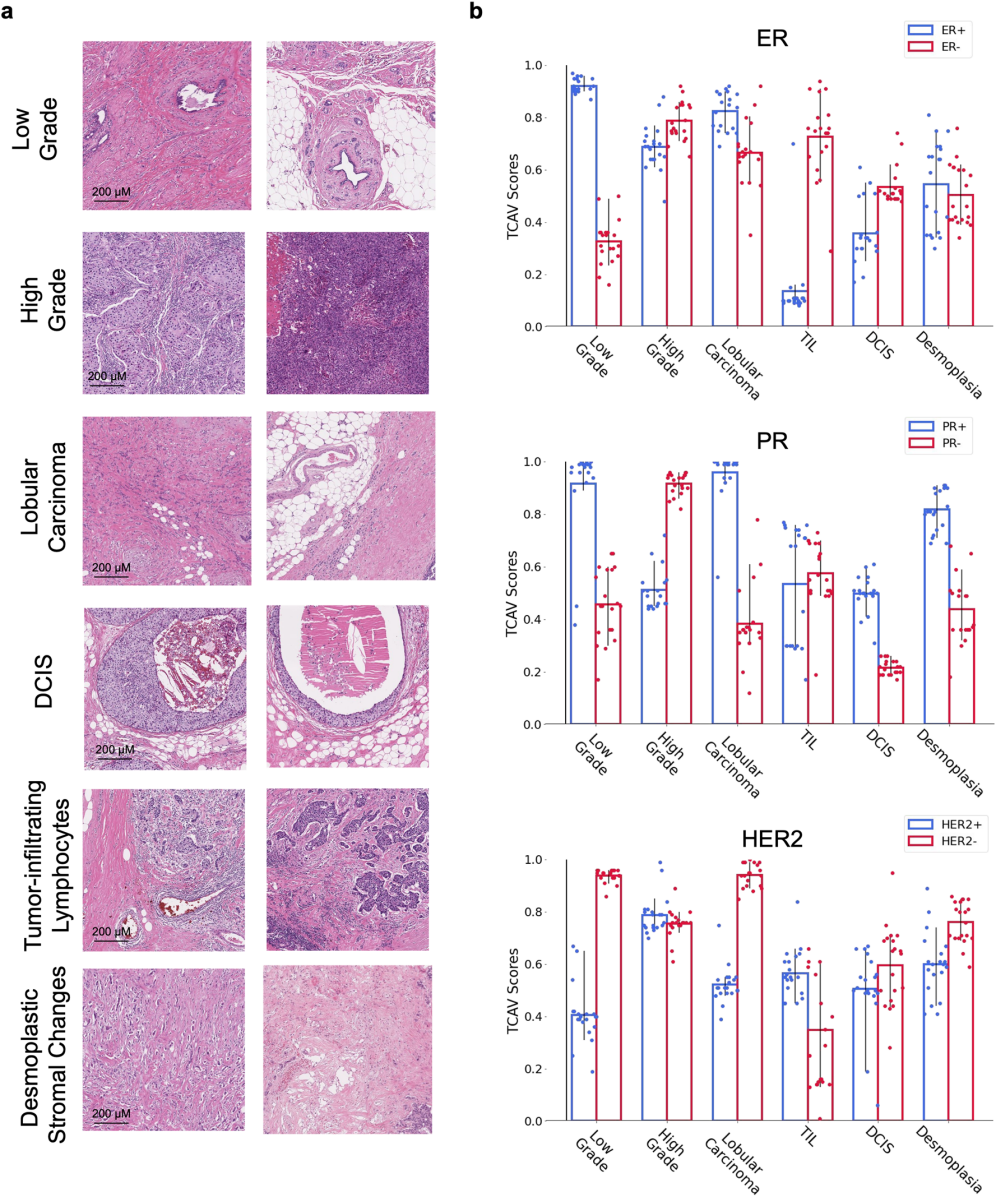
Testing with Concept Activation Vector (TCAV) analysis. **a** Representative concept patches for each of the six concepts used for TCAV analysis. **b** TCAV scores for positive (blue) and negative (red) biomarker status for each of the three biomarkers. Error bars are 95% confidence intervals over 20 trials using 500 class-of-interest patches, 500 random patches, and 100 concept patches per trial. Higher scores indicate stronger association of the concept with the model’s representation of that biomarker status. Detailed TCAV scores are provided in Supplementary Table 3 and Supplementary Data 2. TIL tumor infiltrating lymphocytes, ER Estrogen Receptor, PR Progesterone Receptor, HER2 human epidermal growth factor receptor 2, DCIS Ductal Carcinoma In Situ.

Based on discussion with breast histo-specialists, we selected 6 specific histologic features for which we generated concepts for TCAV analysis: high-grade carcinoma, low-grade carcinoma, invasive lobular carcinoma, ductal carcinoma in-situ (DCIS), tumor-adjacent desmoplastic stromal changes, and tumor infiltrating lymphocytes (TILs) (Fig. 4a). In this analysis, a high TCAV score for a given concept (e.g., high-grade carcinoma) indicates that the specific DLS biomarker prediction is associated with that concept (see Methods for additional details). Figure 4b and Supplementary Table 3 shows each concept’s TCAV score for both positive and negative status predictions for each biomarker. ER-positive predictions were found to be associated with the low-grade concept and ER-negative predictions with TILs. For PR, positive predictions were more strongly associated with low grade, lobular, DCIS, and desmoplasia concepts, while PR-negative predictions were more strongly associated with the high grade concept. For HER2, negative predictions were more strongly associated with low-grade carcinoma and lobular carcinoma concepts.

#### Unsupervised clustering

**Fig. 5:**
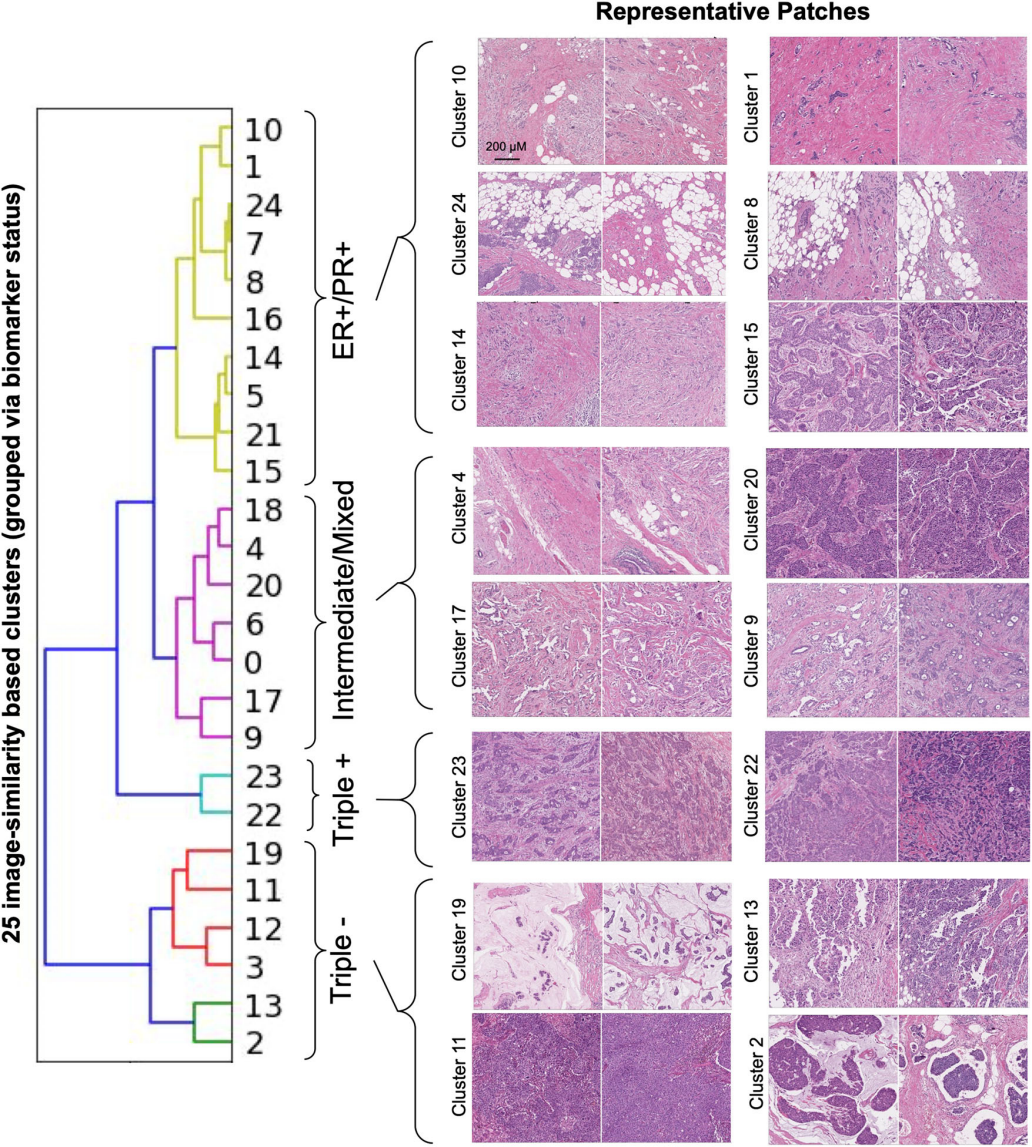
Visualizing similar patches grouped by predicted biomarker status. Sample patches from clusters belonging to each biomarker status prediction grouping are shown. First, patches were clustered based on visual similarity (Cluster 0–24), and then clusters were grouped by applying a second clustering step (hierarchical clustering) using the predicted biomarker status distributions for each cluster (as visualized in the dendrogram). Distributions of biomarker status predictions within each cluster and additional patches for each cluster are shown in Supplementary Fig. 2a–d. Pathologist-provided descriptions of tumor and stromal features for each cluster are summarized in Supplementary Data 1 with additional biomarker prediction and cluster data provided as Supplementary Data 3. For the sample clusters with representative patches in this figure, brief descriptions are as follows: clusters 10 and 1: largely low and intermediate grade tumor with high stromal content; clusters 24 and 8: intermediate grade tumor with diffuse pattern of invasion resulting in a predominant adipose tissue component; cluster 14: intermediate grade tumor with inflammatory cell infiltrates; cluster 15: intermediate grade tumor forming sheets and occasional cribriform morphology; cluster 4: intermediate grade tumor forming cords; clusters 20, 17, and 9: low (17), intermediate (9), and high-grade (20) tumor with variable architecture, occasionally cribriform morphology, and stroma with moderate sclerosis; cluster 23 and 22: intermediate grade, high-tumor-content patches with moderate sclerosis; cluster 19 and 2: low tumor content and presence of extracellular mucin; clusters 11 and 13: high-intermediate grade tumor and high tumor content as well as inflammatory cell infiltrates. Images patches all represent 1024 μM × 1024 μM ER Estrogen Receptor, PR Progesterone Receptor.

Next, to further evaluate associations of histologic features with biomarker predictions in an open-ended manner, we generated 25 clusters of visually similar patches using a deep-learning based clustering approach (Methods). These clusters were then presented to pathologists for histologic characterization (without knowledge of predicted biomarker status). Then, to evaluate the predicted biomarker status for these 25 clusters, we performed hierarchical grouping of the clusters based on the three biomarker prediction scores across each cluster (Methods). This identified four groups of clusters with characteristic biomarker scores: high ER/PR and low HER2 (10 clusters), low ER/PR/HER2 “triple negative” (6 clusters), high ER/PR/HER2 “triple positive” (2 clusters), and “intermediate/mixed” ER/PR/HER2 (7 clusters). These groupings represent the patch-level prediction scores across the cluster and not the final slide-level classification status, for which even small regions of positivity may correspond to positive status. These clusters are further described and shown in Fig. 5, Supplementary Fig. 3a–d, and Supplementary Data 1.

In the first group (high ER/PR and low HER2; Supplementary Fig. 3a), we observed clusters exhibiting largely low and intermediate grade tumor along with high stromal content (clusters 10 and 1), high fat content (clusters 24, 7, 8, and 5), and inflammatory cell infiltrates (clusters 14 and 5). The “triple negative” group (low ER/PR/HER2; Supplementary Fig. 3d) exhibited several clusters with high-intermediate grade tumor and high tumor content as well as inflammatory cell infiltrates (clusters 11, 12, and 13). Additional clusters in this group were notable for low tumor content and presence of extracellular mucin (clusters 2 and 19) or predominant adipose tissue (cluster 3). The third group (high ER/PR/HER2; Supplementary Fig. 3c) consisted of only two clusters, with high-tumor-content patches exhibiting desmoplasia/sclerosis (clusters 22 and 23) and inflammatory cell infiltrates (cluster 22). The last group (Supplementary Fig. 3b) was characterized by substantial variability in model-predicted biomarker status (i.e., the predicted biomarker status was intermediate or mixed, with some patches predicted to be biomarker positive and others negative within a given cluster). This group exhibited largely intermediate grade tumor with variable tumor architecture and stroma. Four of the clusters in this group were noted by pathologists as containing tumor with cribriform morphology (clusters 18, 20, 17, 9).

#### Saliency maps

**Fig. 6:**
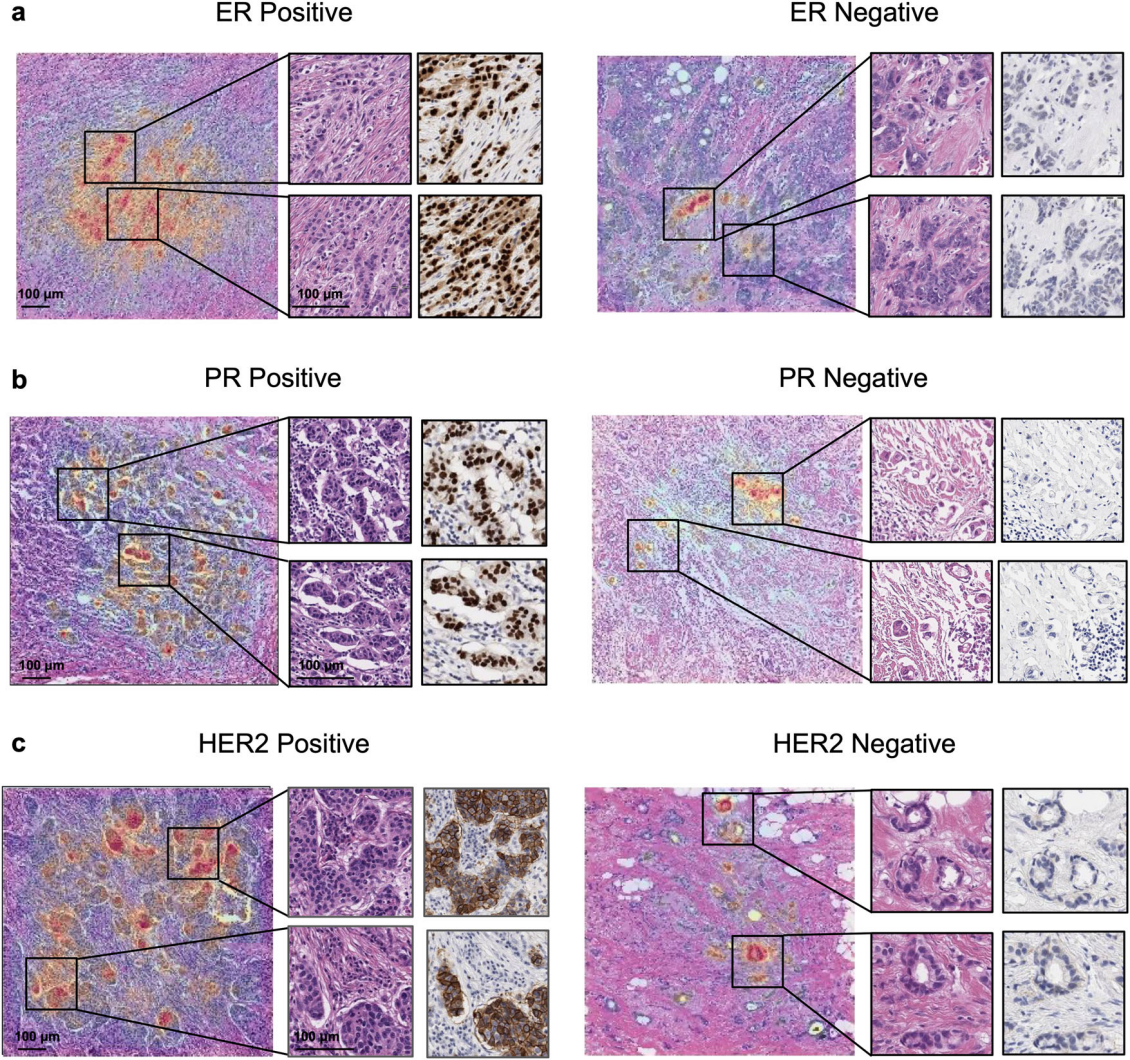
Saliency analysis for patch-level biomarker status prediction. Examples of saliency maps for patches representing each biomarker status prediction; ER (**a**), PR (**b**), and HER2 (**c**). Inset regions highlight higher magnification on both H&E and IHC for the most relevant regions of saliency analysis. Warm colors on the overlay indicate that the underlying pixels from the H&E slide were salient to the model’s predicted probability for the indicated biomarker status. ER Estrogen Receptor, PR Progesterone Receptor, HER2 human epidermal growth factor receptor 2.

Finally, we utilized SmoothGrad^20^ to examine the pixels within each patch that most influenced the patch-level biomarker predictions (Fig. 6). Pathologists reviewed saliency maps for 30 patches randomly selected from each of the positive and negative classes for each biomarker model. For all three biomarkers, saliency maps consistently highlighted tumor cells. Low-grade tumor and linear arrangements of invasive carcinoma cells (consistent with lobular carcinoma) were identified as highly salient elements for ER- and PR-positive predictions. Saliency maps for HER2 positive patches highlighted small clusters of tumor cells as one consistent feature.

## Discussion

Our results demonstrate deep learning-based prediction of ER, PR, and HER2 status directly from histologic features on H&E-stained breast cancer resection specimens. The deep learning models were validated across multiple datasets representing variability in staining and slide preparation. In addition to slide-level predictions, our models provide interpretable, precise predictions of biomarker status for individual tissue regions on H&E slides. These “patch-level” models further enabled multiple interpretability approaches to probe the features associated with the model’s predictions.

Prior reports have described promising results for predicting slide-level biomarker status in breast cancer from H&E images. These works have largely focused on ER prediction, including work on tissue microarray (TMA) datasets^7, 8^ and more recently on whole-slide images utilizing weakly supervised^12^ and unsupervised approaches^9^ for model development. Our findings with a strongly supervised approach further validate the feasibility of slide-level predictions of biomarker status in breast cancer.

In contrast to prior work, however, we adopted a strongly supervised approach for patch-level model training that was enabled by H&E images with paired IHC slides for all three biomarkers. This enables a direct interpretation of models’ regional prediction and demonstrates that it is possible to identify a link between regional histology and the biomarker status. Even if the model prediction for specific regions does not always map perfectly to the IHC stain itself, the observation that heterogeneous predictions corresponds to heterogeneous IHC staining suggests that the models could be used to help identify and evaluate cases with biomarker heterogeneity (Supplementary Fig. 1). Because even a small amount of biomarker positivity can influence the clinical status interpretation (e.g., >1% for ER is considered positive), region-level precision could also be useful to facilitate improved quality control review of IHC by highlighting regions likely to be biomarker positive or negative. Additionally, our patch-level approach enables useful tumor vs. non-tumor segmentation and the potential to distinguish slides containing varying amounts of invasive carcinoma. By contrast, prior weakly supervised approaches do not provide information about the quantity of tumor. As only a single slide is typically selected for IHC, the ability of our model to provide information on both tumor quantity and biomarker status could also improve and potentially help automate the slide selection process for IHC and/or pathologist review. Lastly, while we focused on binary classification as a clinically relevant task, a similar patch-level approach could also be used to develop semi-quantitative models to predict staining intensity and the proportion of positive cells in specific regions. Such models could provide additional utility for flagging equivocal or “low positive” cases and highlighting relevant regions within those slides.

Another important contribution of this work is the utilization of multiple interpretability methods to understand how the models associate morphological features with biomarker status. These specific approaches were enabled by the development of a patch-level model and the results demonstrate human-interpretable associations between morphological features and biomarker status learned by the deep learning model. This work also represents one of the first applications of CAV analysis to histopathology, and demonstrates the opportunity to apply this type of hypothesis-driven feature exploration to other concepts in breast cancer and other deep learning efforts in pathology. The clustering-based analyses utilize semi-quantitative review from multiple pathologists to characterize tumor and stromal features in an unbiased manner, thus expanding on initial qualitative efforts to use clustered visual features^12, 21^.

Specific interpretability findings that models learned known histology-biomarker associations (involving tumor grade, lobular versus ductal type, and TILs) demonstrate that these approaches can highlight relevant features, representing an important step towards building pathologist trust in deep learning approaches. Interestingly, many of these features overlap with those highlighted recently by Naik et al. utilizing a weakly supervised modeling approach and attention-based explainability approach, providing a unique example of the ability to compare features learned via different approaches for a related histopathology task. Other observed associations, such as the enrichment of adipose-predominant patch clusters with ER + /PR + /HER2- predictions or cribriform tumor morphology for clusters with mixed biomarker prediction scores, form a basis for future efforts to explore potentially novel feature-biomarker associations. Also, although the three biomarker models were trained independently, the aggregate predictions provided cluster-groupings that are at least in part consistent with the known subtypes of breast cancer. For example, the cluster-groupings include an “ER-Positive/HER2-negative” group and a “Triple-negative” group, consistent with luminal and basal-like subtypes, respectively. Utilizing deep learning and interpretability techniques to further explore morphological associations with combinations of biomarkers, rather than individual biomarkers, represents another potential application of this type of work.

Nonetheless, not all associations found were consistent with known histologic-biomarker relationships. For example, the finding that patches from mucinous tumors (e.g., cluster 19) were predominately classified as ER/PR negative does not align with studies which find these tumors to be mostly ER/PR positive^22^. This may be due in part to limited training data for mucinous tumors as well as the fact that IHC staining itself may be variable in mucinous patches. Relatedly, as the cases used for this study were not selected or enriched for any particular invasive breast carcinoma subtype, the training dataset will inherently consist of the most common histologic subtypes and morphologic features. Developing and evaluating biomarker models for specific histologic subtypes of breast cancer may also be useful and may result in different or additional insights and learned features.

While our models appear to have primarily learned known feature-biomarker associations, we envision hypothesis-driven TCAV analysis along with hypothesis-generating cluster analysis can form a useful interpretability framework for identifying both known and unknown features learned by AI models in pathology. This raises interesting questions of both feasibility and trust in regard to identification of known versus unknown features learned by a model. A key challenge for any efforts aimed at discovering novel features is avoiding the inherent bias of humans towards “seeing” and describing only the known features. Utilizing complementary approaches and obtaining “blinded” input from multiple experts may represent an initial step in addressing this challenge, but creative approaches to incorporate machine learning feature extraction and pathologist expertise will be required given that some learned features may not be identified by visual review alone. Any novel features or associations that are identified will also require validation and proven reliability, just as for the known biomarker-feature associations that have been established over time.

Our work does have some limitations. In regards to slide level evaluation, the historical biomarker statuses (sampled and tested between 1988 and 2016) are only provided at the case-level (without reference to the specific slide or block used for IHC evaluation). By applying case labels to every slide within a given case, we may be training or evaluating using noisy slide-level biomarker labels in instances of heterogeneous biomarker expression across slides in a case. While we expect such within-case heterogeneity to affect only a small portion of cases, this issue does not impact the IHC-based patch-level training and evaluation, further highlighting the value of this aspect of model development as an important contribution of the present work. Additionally, technical and interpathologist variability impacting interpretation of IHC may also impact annotations and historically reported biomarker status, particularly for the TCGA dataset because it includes data from multiple institutions. Of note, interobserver variability is likely to be a more substantial issue for HER2 than for ER and PR^23–25^, and as such, the lower model performance for HER2 may in part reflect a less reliable ground truth for this particular biomarker, especially those based on historical clinical reports. IHC protocols and interpretation guidelines have also changed over time, further contributing to potential variability across historical clinical labels. Still, the relatively lower performance for the HER2 models warrant future exploration to understand if associated feature diversity, relative proportion of positive and equivocal cases, or other factors contribute, and if potentially larger training data sets can overcome these challenges. Future work using IHC-based labels for both slides and cases, complete clinical slide sets, and prospective studies all represent valuable steps towards clinical validation and implementation. Lastly, demographic data like race, ethnicity, or age were not consistently available in our datasets, limiting subgroup analysis of the model’s performance. Further validation across demographically diverse cohorts is required.

In summary, this study demonstrates generalizable deep learning models for predicting ER, PR, and HER2 status in breast cancer from H&E images and expands upon the growing body of literature for rapid biomarker estimation from routine histology slides. While further performance improvement and validation is still needed before automated breast cancer biomarker prediction models find their way to clinical workflows, initial utility may also be realized via research and quality control applications. Specifically, biomarker-based selection or triage of patients within large clinical trials could create substantial efficiency gains for therapy development pipelines. Automated biomarker interpretations could also supplement IHC workflows by identifying equivocal cases for appropriate follow-up evaluation or flagging potential technical issues based on discordant IHC and model predictions. Furthermore, this approach could help identify heterogeneous tumors or to select the most informative tissue blocks for biomarker evaluation. Lastly, interpretability methods for identifying histologic features associated with biomarker status could guide researchers to investigate new biological mechanisms and molecular targets related to the underlying morphologic findings.

### Reporting summary

Further information on research design is available in the Nature Research Reporting Summary linked to this article.

## Supplementary information

Supplementary Materials

Supplementary Data 1

Supplementary Data 2

Supplementary Data 3

Supplementary Data 4

Description of Additional Supplementary Files

Reporting Summary

## Data Availability

Source Data for the main figures in the manuscript with statistical analyses are provided in Supplementary Data files 1–4. TCGA data utilized in this study corresponds to the Breast Invasive Carcinoma (BRCA) study from TCGA and is publicly available via the Genomic Data Commons Data Portal (gdc.cancer.gov). The tertiary hospital dataset was used under a Defense Health Agency data sharing agreement. Requests regarding data can be directed to the Defense Health Agency Privacy Office at DHA.PrivacyOfficeMail@mail.mil. The medical laboratory dataset is not publicly available at this time due to data privacy considerations but may be available from the corresponding author on reasonable request.

## Code availability

The deep learning framework (TensorFlow v1.14) used in this study is available at https://github.com/tensorflow/tensorflow/tree/r1.14. The convolutional neural network Inception-V3^16^ is available at https://github.com/tensorflow/models/blob/master/research/slim/nets/inception_v3.py. The stage 2 model was implemented with scikit-learn (v0.24.0) and available at https://github.com/scikit-learn/scikit-learn/tree/0.24.X. The saliency analysis library (v0.0.6) is available at https://github.com/PAIR-code/saliency. The TCAV analysis library (v0.2.1) is available at https://github.com/tensorflow/tcav/tree/0.2. The final, trained models have not yet undergone regulatory review and cannot be made available at this time. Interested researchers can contact P.-H.C.C. for questions on its status and access.
