# Supplementary material for: Determining breast cancer biomarker status and associated morphological features using deep learning
Source: Commun Med (Lond). 2021 Jul 14;1:14. doi: 10.1038/s43856-021-00013-3 (PMC9037318; doi:10.1038/s43856-021-00013-3)
Supplement: Supplementary file 1 — Supplementary Materials [file 43856_2021_13_MOESM1_ESM.pdf]

Determining Breast Cancer Biomarker Status and Associated Morphological Features Using Deep Learning

Authors

Paul Gamble, Ronnachai Jaroensri, Hongwu Wang, Fraser Tan, Melissa Moran, Trissia Brown, Isabelle Flament-Auvigne, Emad A. Rakha, Michael Toss, David J. Dabbs, Peter Regitnig, Niels Olson, James H. Wren, Carrie Robinson, Greg S. Corrado, Lily H. Peng, Yun Liu, Craig H. Mermel, David F. Steiner, Po-Hsuan Cameron Chen

Supplementary Information

Supplementary Methods

Alignment of H&E and IHC slides

We utilized a rigid transformation to align the pairs of gigapixel H&E and IHC images, with the parameters of the transformation obtained via a 2-step (coarse-to-fine) process. For the coarse-grained alignment, we generated coarse-grained image features by applying CONGAS[25] at “0.3125X magnification” (i.e., a whole-slide image that was digitized at 20X would be downsampled by a factor of 64). Next, we used random sample consensus (RANSAC)[26] to compute a coarse alignment using these features. For the fine-grained alignment, we refined the matched features at a high magnification (40X) using a template-matching method, and used RANSAC again to compute the final rigid transformation.

TCGA data and slide-level labels

All 1122 available TCGA BRCA study FFPE images were reviewed by one pathologist per image. Slides deemed to have poor image or stain quality, tissue processing artifact, or absence of invasive breast carcinoma were removed (n=138). Status for ER, PR, and HER2 were obtained from available TCGA data via the Genomic Data Commons portal as well as associated TCGA publications[13,14]. Additional information about TCGA can be found at http://cancergenome.nih.gov. The available pathology reports were also reviewed and biomarker status was manually extracted when available. Cases for which biomarker status in the clinical notes was discordant with the status in the structured TCGA data (positive in one, negative in the other) were excluded from analysis for that biomarker (ER: n=14, PR: n=28, HER2: n=14).

Invasive carcinoma segmentation

Our patch classification models are trained to distinguish between three classes: biomarker positive invasive carcinoma, biomarker negative invasive carcinoma, and “other” (i.e., tissue that is not invasive carcinoma, including DCIS). By combining the first two classes into a single invasive carcinoma class, we can assess the performance of our models at differentiating between tumor and non-tumor. The patch-level AUC for detecting invasive carcinoma vs. “other” (regardless of biomarker status) was 0.974 (95%CI 0.972-0.976), 0.965 (95%CI 0.963-0.967), and 0.944 (95%CI 0.941-0.947) for the ER, PR, and HER2 models, respectively.

Concept Activation Vector (CAV) Analysis Details

To conduct concept activation vector (CAV) analysis, we first generated the embeddings (i.e., the activations of the concatenation layer immediately before the final fully connected layers of

our DLS) for all concept patches and random patches. For each concept, we then trained a set of 20 linear support vector machine (SVM) classifiers to distinguish between activations of all concept patches and random patches. These SVMs are trained with hinge loss, L2 regularization with alpha = 0.0001, and 1000 iterations with an early stopping tolerance of 0.001. The CAV for each concept is defined as the vector orthogonal to the separating hyperplane of its corresponding classifier. Next, the sensitivity of the DLS's predictions to each concept is measured by computing the directional derivative of the prediction along the CAV.

Positive directional derivatives indicate that patches that are more likely to be classified as belonging to the given class if they had activations that were slightly more similar to the concept. Concretely - if the ER Positive class probability of a random patch sampled from a known tumor region has a positive directional derivative in activation space along the Concept Activation Vector for “low grade carcinoma”, this indicates that if that particular patch was altered such that it was represented by the model as slightly more similar to known low grade patches, it would be slightly more likely to be classified as ER Positive. For each concept-biomarker pair, we report the “testing with CAV” (TCAV) score, which is defined as the fraction of patches of a known class that have a positive derivative, with confidence intervals computed across the 20 samples.[15] For many random patches, the fraction of positive directional derivatives gives an indication of the association formed by a model between a concept and a predicted class, particularly when compared to another class within the same model.

Supplementary Tables

Supplementary Table 1. Hyperparameters for ER, PR, HER2 Patch Classification Models

Network configuration	Architecture: Inception v3<br>Depth multiplier: 0.2<br>L2 weight decay: 4e-05<br>Batch norm decay rate: 0.99<br>Loss function: softmax cross-entropy<br>Ensemble size: 10 models (random initialization)<br>Ensembling averaging method: geometric mean
Model Inputs	Magnification: 5X (2.4 μm/pixel)<br>Patch size: 512x512<br>Stain normalization applied using reference color statistics from a fixed slide.
Data augmentation	Label propagation across serial sections (see Methods)<br>Orientation randomization: left/right mirroring and all 4 rotations<br>Brightness: tf.image.random_brightness with max_delta=0.25<br>Saturation: tf.image.random_saturation with lower=0.75 and upper=1.25<br>Hue: tf.image.random_hue with max_delta=0.04<br>Contrast: tf.image.random_contrast with lower=0.25 and upper=1.75
Training	Batch size: 32<br>Training steps: 6,000,000
Optimizer configuration	Optimizer: RMSProp<br>Decay: 0.9<br>Epsilon: 1<br>Momentum: 0.9
Learning Rate	Initial: 0.0055<br>Exponential decay: 0.9<br>Decay steps: 200000

Supplementary Table 2. Tumor subtype distribution across test sets.

	DLS Stage 1 Test Set (patch-level)	DLS Stage 2 Test Set (slide-level)	
Dataset Source	Tertiary Teaching Hospital	Tertiary Teaching Hospital	TCGA
No. of cases	64	340	909
No. of H&E slides	181	2,313	961
Num. of Ductal Carcinoma Cases	57	292	651
Num. of Lobular Carcinoma Cases	4	32	169
Num. of Mucinous Carcinoma Cases	0	13	15
Num. of Not Specified, Mixed and other Cases	3	3	74

Supplementary Table 3. Quantitative results of TCAV interpretability results for each biomarker.

	ER Positive	ER Negative
Low Grade	0.92 (95% CI 0.90-0.96)	0.33 (95% CI 0.24-0.49)
High Grade	0.69 (95% CI 0.61-0.77)	0.79 (95% CI 0.71-0.86)
Lobular	0.83 (95% CI 0.74-0.90)	0.67 (95% CI 0.55-0.81)
TILs	0.14 (95% CI 0.09-0.16)	0.73 (95% CI 0.58-0.91)
DCIS	0.36 (95% CI 0.25-0.55)	0.54 (95% CI 0.49-0.64)
Desmoplasia	0.55 (95% CI 0.34-0.75)	0.50 (95% CI 0.39-0.62)

	PR Positive	PR Negative
Low Grade	0.92 (95% CI 0.89-1.0)	0.46 (95% CI 0.29-0.6)
High Grade	0.51 (95% CI 0.44-0.62)	0.91 (95% CI 0.86-0.97)
Lobular	0.96 (95% CI 0.92-1.0)	0.38 (95% CI 0.31-0.64)
TILs	0.54 (95% CI 0.29-0.76)	0.58 (95% CI 0.49-0.70)
DCIS	0.50 (95% CI 0.41-0.60)	0.22 (95% CI 0.19-0.26)
Desmoplasia	0.81 (95% CI 0.71-0.92)	0.44 (95% CI 0.32-0.59)

	HER2 Positive	HER2 Negative
Low Grade	0.41 (95% CI 0.31-0.65)	0.94 (95% CI 0.91-0.97)
High Grade	0.79 (95% CI 0.73-0.85)	0.75 (95% CI 0.72-0.81)
Lobular	0.54 (95% CI 0.48-0.56)	0.94 (95% CI 0.89-0.99)
TILs	0.57 (95% CI 0.45-0.67)	0.35 (95% CI 0.14-0.61)
DCIS	0.51 (95% CI 0.20-0.66)	0.60 (95% CI 0.43-0.71)
Desmoplasia	0.60 (95% CI 0.44-0.74)	0.76 (95% CI 0.69-0.85)

Supplementary Figures

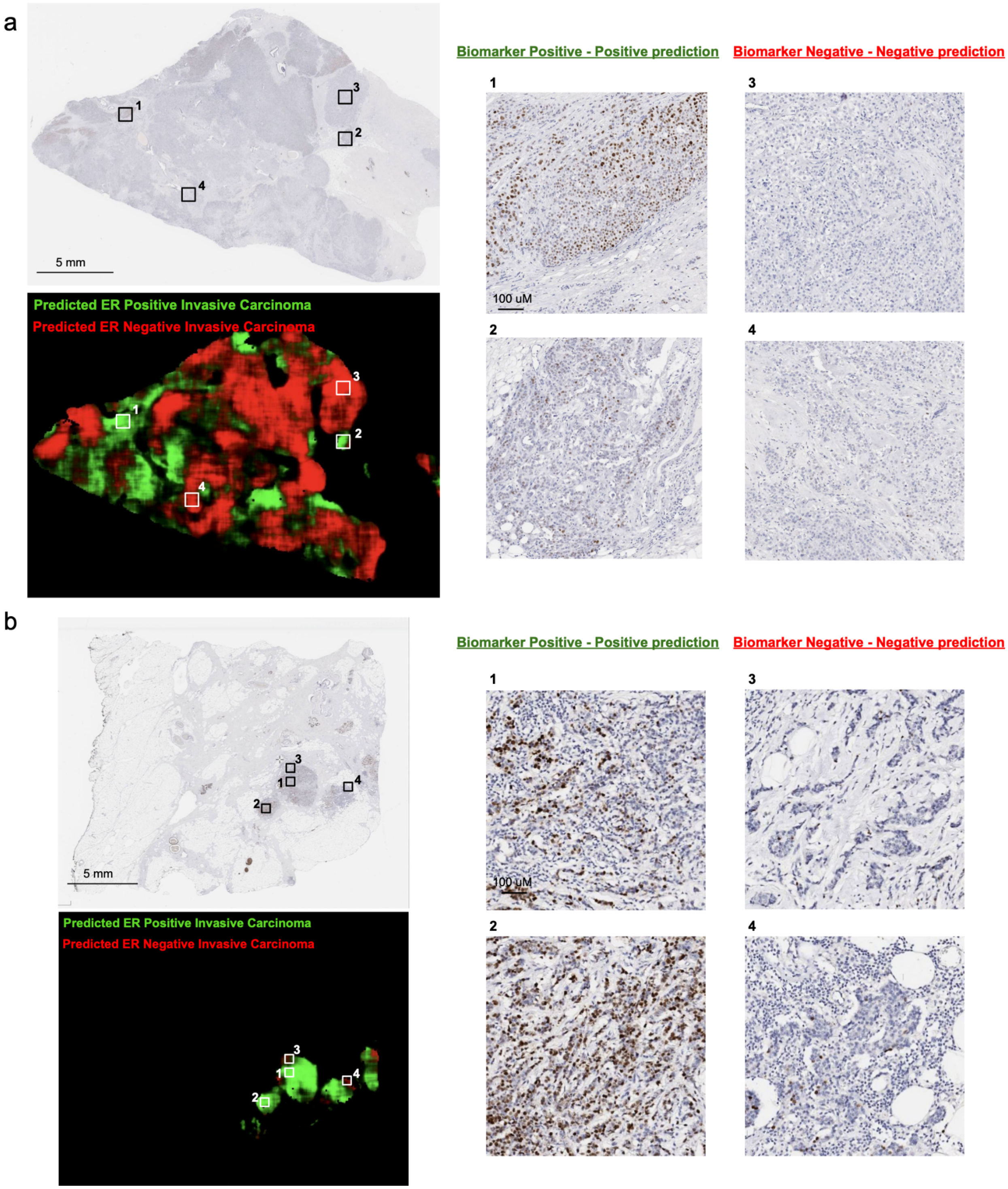

Supplementary Figure 1. Visualization of ER prediction heatmaps for slides with heterogeneous ER expression. Immunohistochemistry (IHC) slides for ER and the corresponding heatmap predictions are shown for two independent slides from different cases, (a) and (b). Sampled regions are shown at higher magnification as indicated for reference. The patch-level prediction heatmaps were produced by running inference of the ER stage 1 model on the corresponding H&E slides. Regions predicted to be ER positive invasive carcinoma by the model are shown in green and regions predicted to be ER negative are shown in red. Only regions predicted to be invasive carcinoma are highlighted. These slides exhibit patchy or heterogenous ER expressions based on IHC and pathologist review, as also reflected by the heterogenous ER status predictions by the model for these slides.

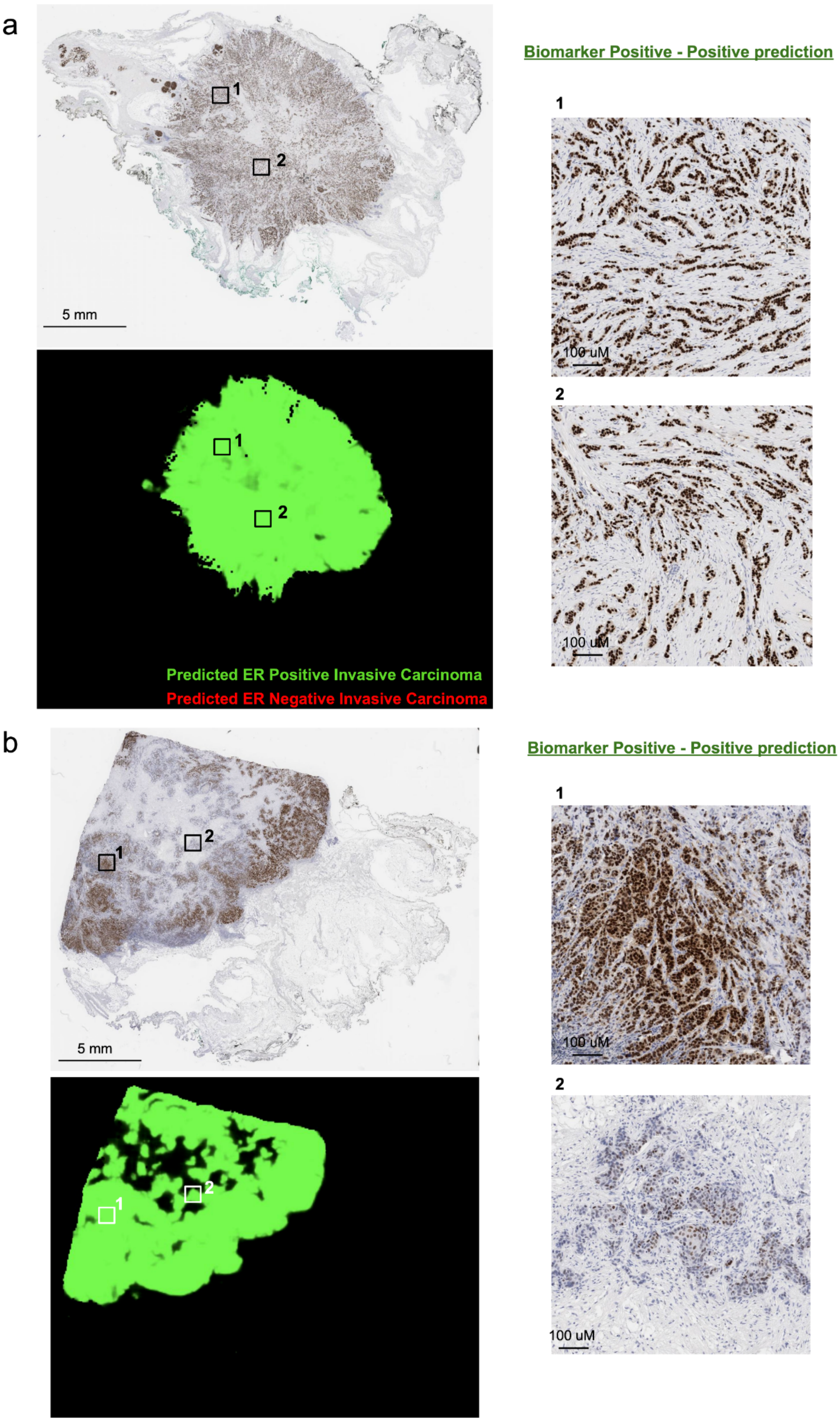

a
1
2
5 mm
1
2
Predicted ER Positive Invasive Carcinoma
Predicted ER Negative Invasive Carcinoma
Biomarker Positive - Positive prediction
1
100 uM
2
100 uM
b
1
2
5 mm
1
2
Biomarker Positive - Positive prediction
1
100 uM
2
100 uM

Supplementary Figure 2. Visualization of ER prediction heatmaps for slides with homogenous ER positivity. Immunohistochemistry (IHC) slides for ER and the corresponding heatmap predictions are shown for two independent slides from different cases, (a) and (b). Sampled regions are shown at higher magnification as indicated for reference. The patch-level prediction heatmaps were produced by running inference of the ER stage 1 model on the corresponding H&E slides. These slides exhibit ER expression for the majority of tumor cells based on IHC and pathologist review, as also reflected by the homogenous, positive ER status predictions by the model for these slides.

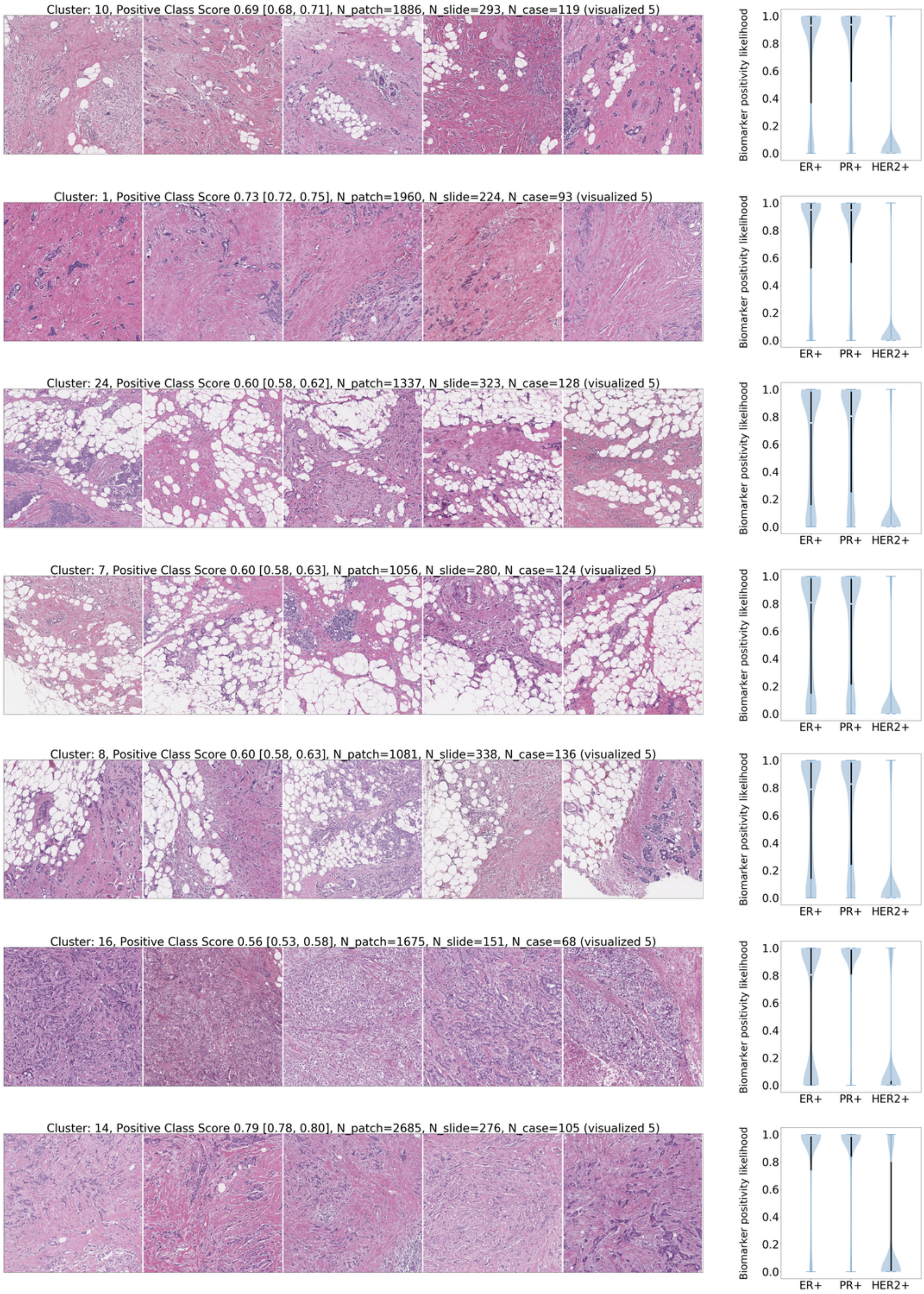
Cluster: 10, Positive Class Score 0.69 [0.68, 0.71], N_patch=1886, N_slide=293, N_case=119 (visualized 5)
Cluster: 1, Positive Class Score 0.73 [0.72, 0.75], N_patch=1960, N_slide=224, N_case=93 (visualized 5)
Cluster: 24, Positive Class Score 0.60 [0.58, 0.62], N_patch=1337, N_slide=323, N_case=128 (visualized 5)
Cluster: 7, Positive Class Score 0.60 [0.58, 0.63], N_patch=1056, N_slide=280, N_case=124 (visualized 5)
Cluster: 8, Positive Class Score 0.60 [0.58, 0.63], N_patch=1081, N_slide=338, N_case=136 (visualized 5)
Cluster: 16, Positive Class Score 0.56 [0.53, 0.58], N_patch=1675, N_slide=151, N_case=68 (visualized 5)
Cluster: 14, Positive Class Score 0.79 [0.78, 0.80], N_patch=2685, N_slide=276, N_case=105 (visualized 5)
Biomarker positivity likelihood
0.0
0.2
0.4
0.6
0.8
1.0
ER+
PR+
HER2+

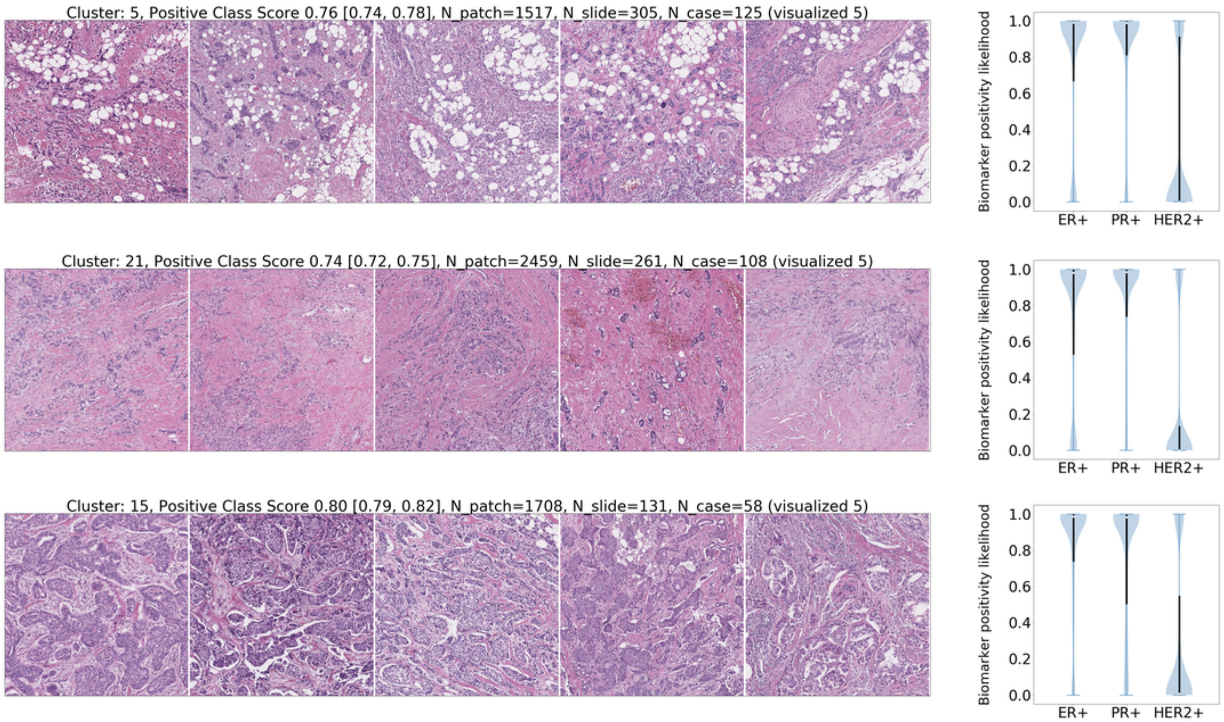

Supplementary Figure 3a. Example patches for the “ER+ / PR+” clusters. Random patches from each cluster are shown. Violin plots represent biomarker prediction distribution for individual patches over all patches in the corresponding cluster. Interquartile range and median biomarker likelihood scores for each cluster are available in Supplementary Data 3. Patches are 512 pixels x 512 pixels at 5x magnification (1024 uM).

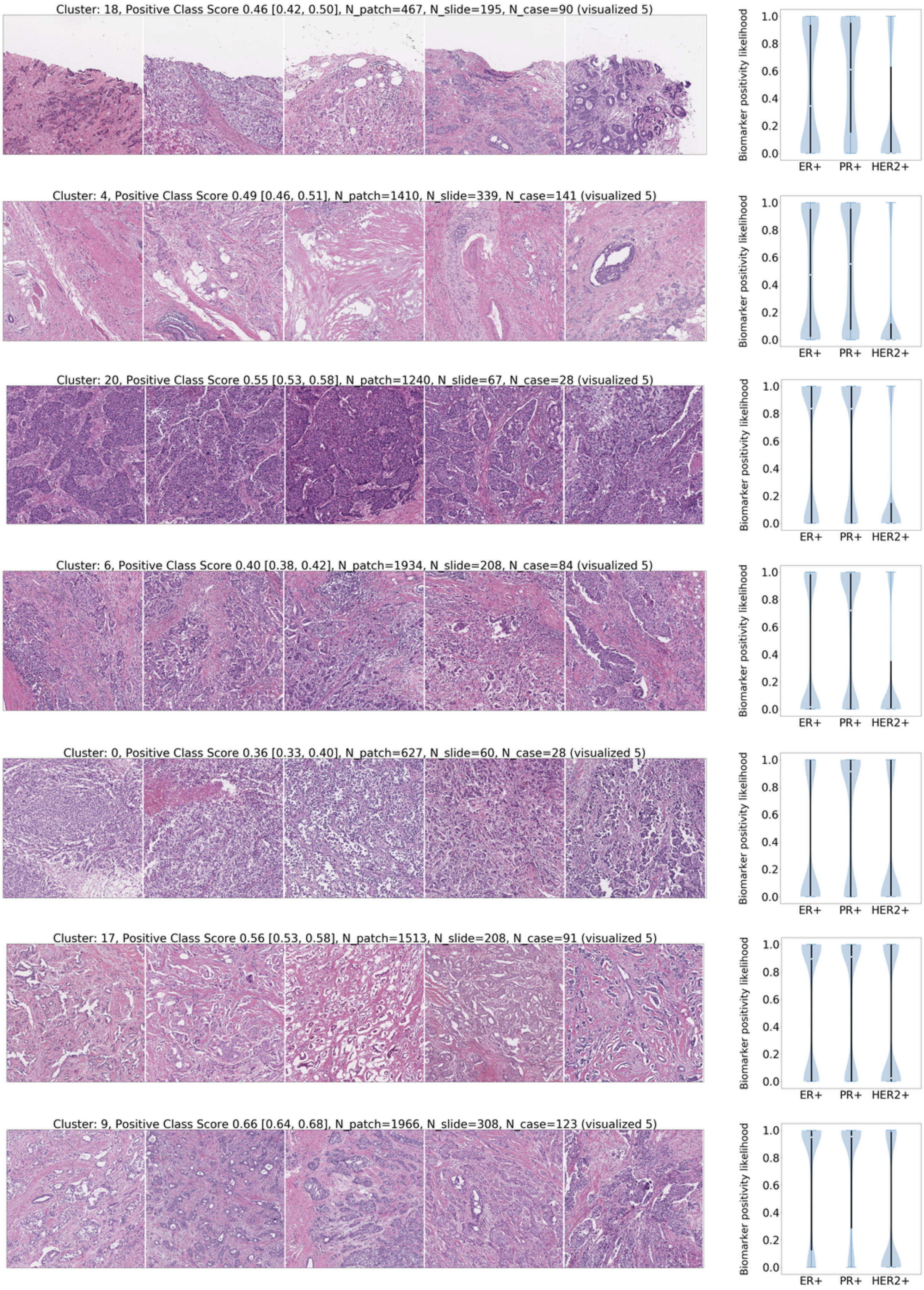

Supplementary Figure 3b. Example patches for the "Intermediate/Mixed" clusters. Random patches from each cluster are shown. Violin plots represent biomarker prediction distribution for

individual patches over all patches in the corresponding cluster. Interquartile range and median biomarker likelihood scores for each cluster are available in Supplementary Data 3. Patches are 512 pixels x 512 pixels at 5x magnification (1024 uM).

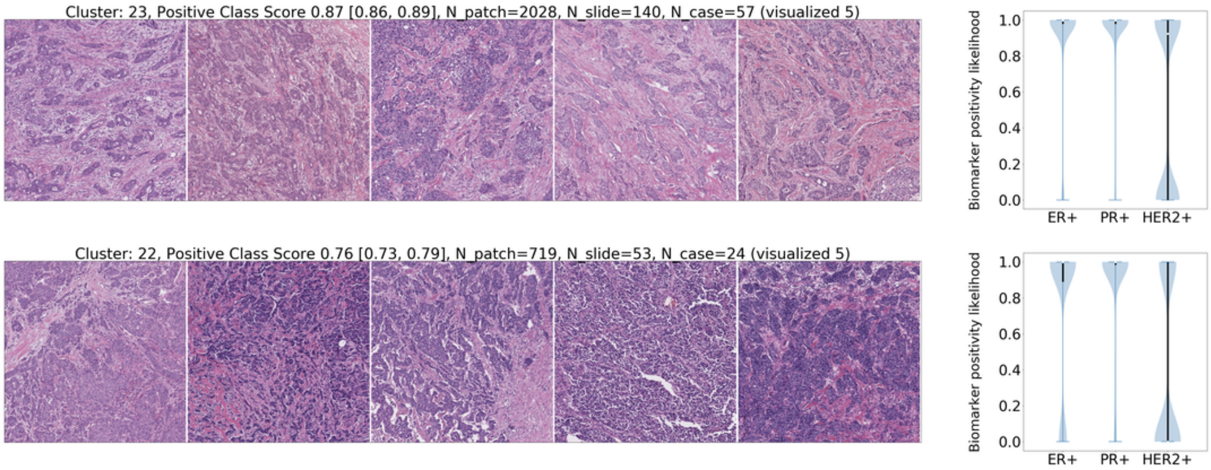

Supplementary Figure 3c. Example patches for the "Triple Positive" clusters. Random patches from each cluster are shown. Violin plots represent biomarker prediction distribution for individual patches over all patches in the corresponding cluster. Interquartile range and median biomarker likelihood scores for each cluster are available in Supplementary Data 3. Patches are 512 pixels x 512 pixels at 5x magnification (1024 uM).

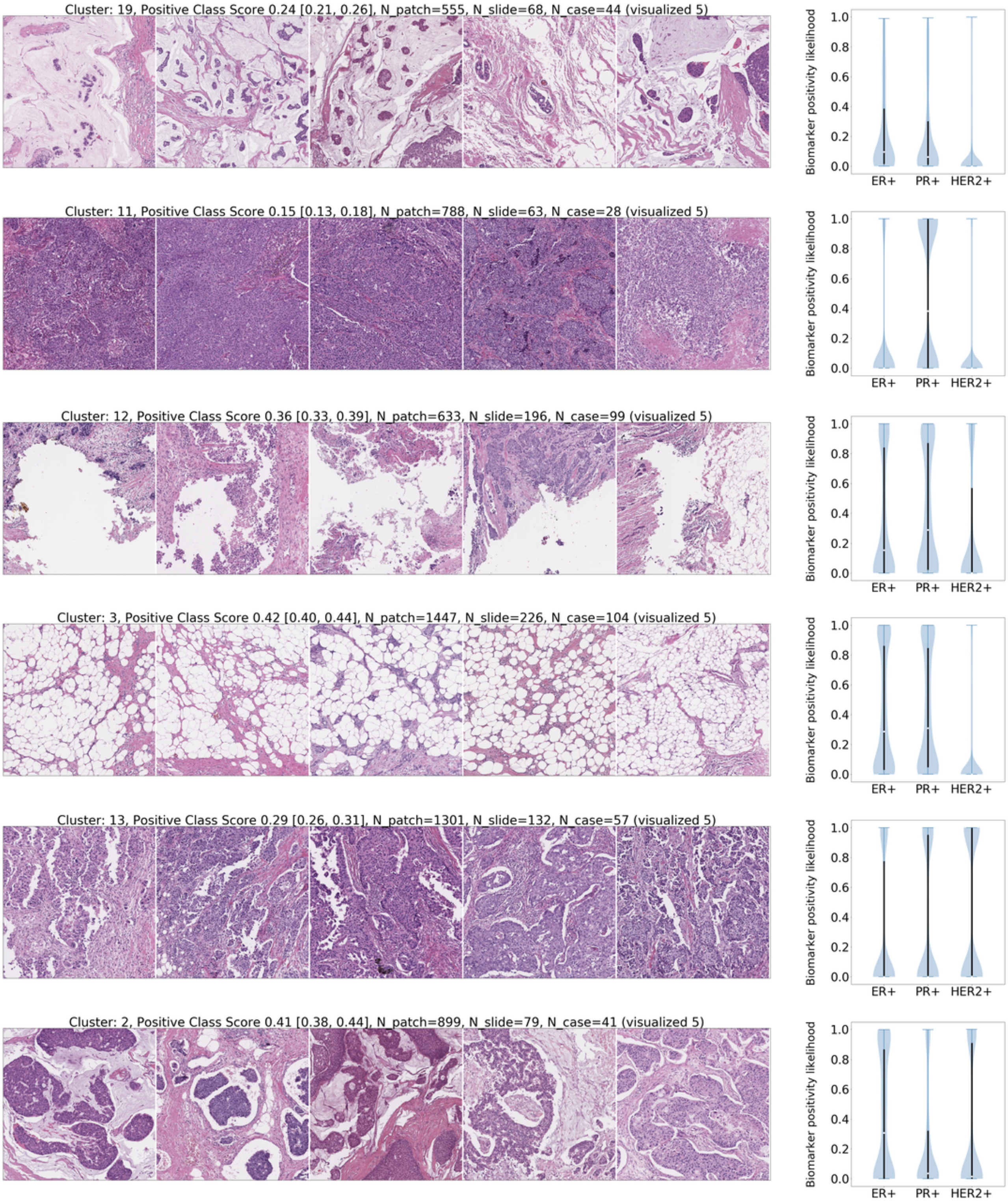

Supplementary Figure 3d. Example patches for the "Triple Negative" clusters. Random patches from each cluster are shown. Violin plots represent biomarker prediction distribution for individual patches over all patches in the corresponding cluster. Interquartile range and median biomarker likelihood scores for each cluster are available in Supplementary Data 3. Patches are 512 pixels x 512 pixels at 5x magnification (1024 uM).

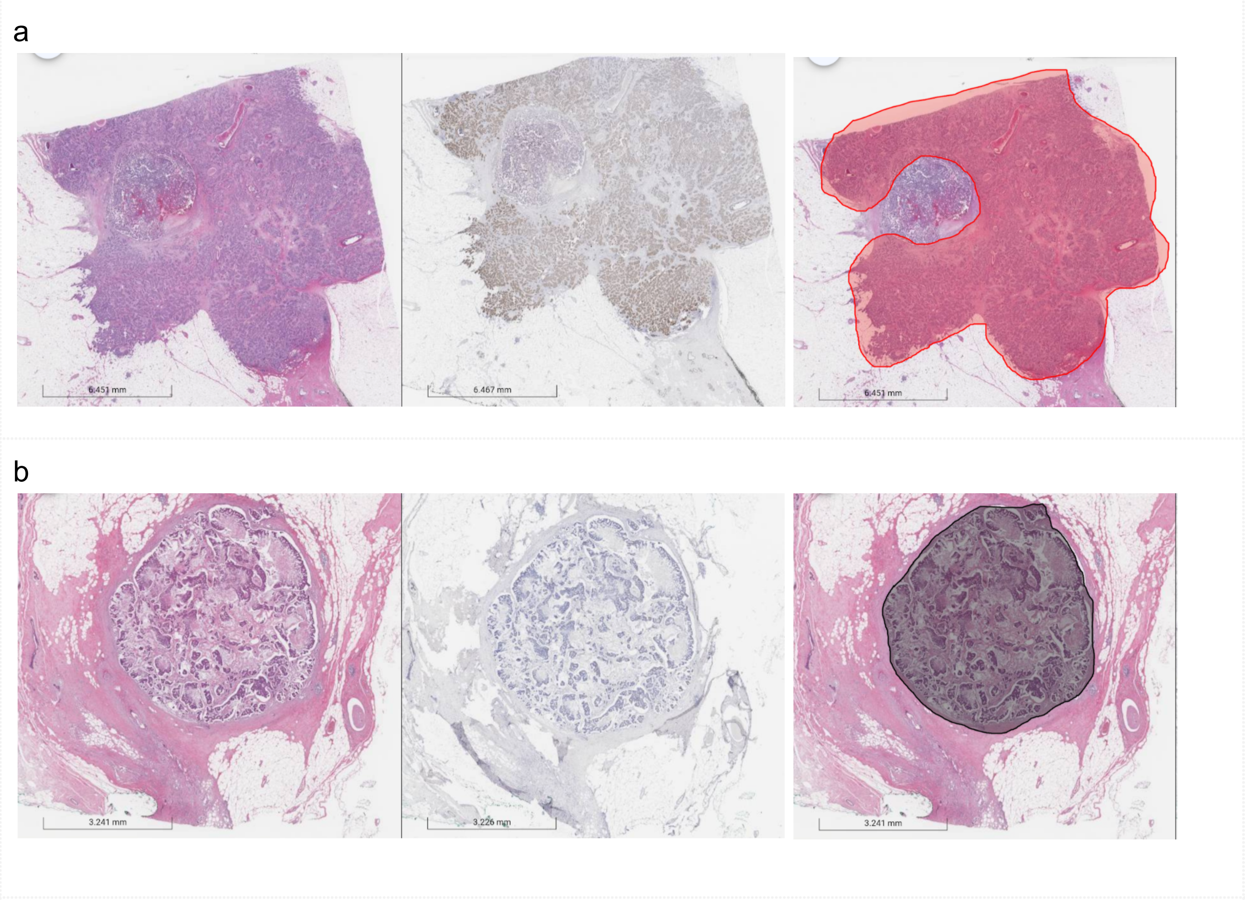

Supplementary Figure 4. Overview of input data and labels. Example WSIs for H&E image (left), IHC image (middle), and expected annotation (right). (**a**) Example of biomarker-positive invasive carcinoma. (**b**) Example of a biomarker-negative invasive carcinoma annotation.

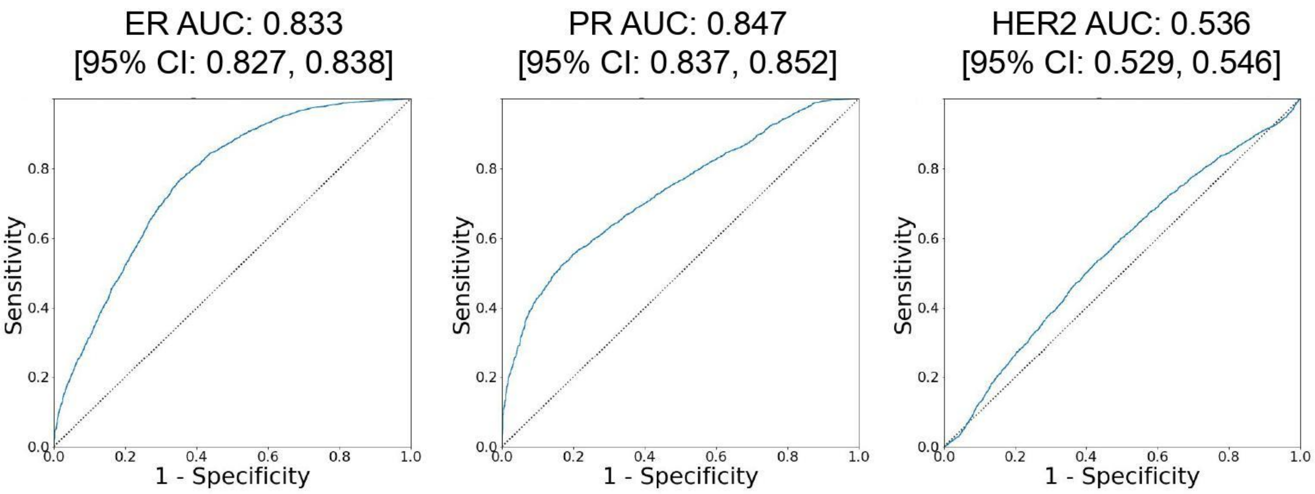

Supplementary Figure 5. Patch-level ROC curves considering only invasive carcinoma (positive and negative) for each biomarker. The number of patches used to evaluate these models are available in Table 1b.
