# Supplementary material for: Determining breast cancer biomarker status and associated morphological features using deep learning
Source: Commun Med (Lond). 2021 Jul 14;1:14. doi: 10.1038/s43856-021-00013-3 (PMC9037318; doi:10.1038/s43856-021-00013-3)
Supplement: Supplementary file 6 — Description of Additional Supplementary Files [file 43856_2021_13_MOESM6_ESM.pdf]

Description of Additional Supplementary Files

File Name: Supplementary Data 1

Description: Image Cluster summary organized by hierarchical grouping Summary of cluster characterization by pathologists. Cluster colors correspond to the hierarchical grouping and median biomarker prediction scores for each cluster are color coded by value (0-1).

File Name: Supplementary Data 2

Description: TCAV analysis

File Name: Supplementary Data 3

Description: Biomarker prediction clustering.

File Name: Supplementary Data 4

Description: ROC curve analysis data. Data for each panel of Figure 2 is provided as a separate tab in the data file.
