# Supplementary material for: Determining breast cancer biomarker status and associated morphological features using deep learning
Source: Commun Med (Lond). 2021 Jul 14;1:14. doi: 10.1038/s43856-021-00013-3 (PMC9037318; doi:10.1038/s43856-021-00013-3)
Supplement: Supplementary file 7 — Reporting Summary [file 43856_2021_13_MOESM7_ESM.pdf]

nature research

Corresponding author(s): David Steiner
Cameron Chen

Last updated by author(s): Jun 8, 2021

Reporting Summary

Nature Research wishes to improve the reproducibility of the work that we publish. This form provides structure for consistency and transparency in reporting. For further information on Nature Research policies, see our Editorial Policies and the Editorial Policy Checklist.

Statistics

For all statistical analyses, confirm that the following items are present in the figure legend, table legend, main text, or Methods section.

n/a	Confirmed	
☐	☒	The exact sample size (n) for each experimental group/condition, given as a discrete number and unit of measurement
☒	☐	A statement on whether measurements were taken from distinct samples or whether the same sample was measured repeatedly
☐	☒	The statistical test(s) used AND whether they are one- or two-sided<br>*Only common tests should be described solely by name; describe more complex techniques in the Methods section.*
☒	☐	A description of all covariates tested
☐	☒	A description of any assumptions or corrections, such as tests of normality and adjustment for multiple comparisons
☐	☒	A full description of the statistical parameters including central tendency (e.g. means) or other basic estimates (e.g. regression coefficient) AND variation (e.g. standard deviation) or associated estimates of uncertainty (e.g. confidence intervals)
☐	☒	For null hypothesis testing, the test statistic (e.g. F, t, r) with confidence intervals, effect sizes, degrees of freedom and P value noted<br>*Give P values as exact values whenever suitable.*
☒	☐	For Bayesian analysis, information on the choice of priors and Markov chain Monte Carlo settings
☒	☐	For hierarchical and complex designs, identification of the appropriate level for tests and full reporting of outcomes
☒	☐	Estimates of effect sizes (e.g. Cohen's d, Pearson's r), indicating how they were calculated

Our web collection on statistics for biologists contains articles on many of the points above.

Software and code

Policy information about availability of computer code

Data collection	Internal slide viewing and annotation software was used for annotation collection, otherwise no software used for data collection. Please see datasets section of methods for additional details of data collection. All available slides/images were reviewed by pathologists for quality control and the inclusion criteria of invasive carcinoma in primary breast tissue specimens. Status for ER, PR, and HER2 were obtained from available TCGA data via the Genomic Data Commons portal as well as associated TCGA publications. For both TCGA and non-TCGA data, The available pathology reports were also reviewed and biomarker status was manually extracted when available. Cases for which biomarker status in the clinical notes was discordant with the status in the structured TCGA data (positive in one, negative in the other) were excluded from analysis for that biomarker.
Data analysis	The open-sourced library TensorFlow was used to develop the models, and the scikit-learn library and custom Python code was used for statistical analysis. The libraries used for saliency analysis and TCAV analysis are available at https://github.com/PAIR-code/saliency and https://github.com/tensorflow/tcav, respectively.

For manuscripts utilizing custom algorithms or software that are central to the research but not yet described in published literature, software must be made available to editors and reviewers. We strongly encourage code deposition in a community repository (e.g. GitHub). See the Nature Research guidelines for submitting code & software for further information.

Data

Policy information about availability of data

All manuscripts must include a data availability statement. This statement should provide the following information, where applicable:

- Accession codes, unique identifiers, or web links for publicly available datasets
- A list of figures that have associated raw data
- A description of any restrictions on data availability

Source Data for the main figures in the manuscript with statistical analyses are provided in Supplementary Data files 1-4. TCGA data utilized in this study corresponds to the Breast Invasive Carcinoma (BRCA) study from TCGA and is publicly available via the Genomic Data Commons Data Portal (gdc.cancer.gov). The tertiary hospital dataset was used under a Defense Health Agency data sharing agreement. Requests regarding data can be directed to the Defense Health Agency Privacy Office at DHA.PrivacyOfficeMail@mail.mil. The medical laboratory dataset is not publicly available at this time due to data privacy considerations but may be available from the corresponding author on reasonable request.

Field-specific reporting

Please select the one below that is the best fit for your research. If you are not sure, read the appropriate sections before making your selection.

☒ Life sciences ☐ Behavioural & social sciences ☐ Ecological, evolutionary & environmental sciences

For a reference copy of the document with all sections, see nature.com/documents/nr-reporting-summary-flat.pdf

Life sciences study design

All studies must disclose on these points even when the disclosure is negative.

Sample size	Based on data availability from the included data sources. For the train-test setup of this algorithm development, evaluation on the test set validates sufficiency of sample size used for training.
Data exclusions	Quality control review of images and completeness of associated biomarker status.
Replication	Two validation sets, including an internal held out, random split from the data source used for model development and an external set (TCGA) not used for model development
Randomization	Random split for train, tune, test sets from development datasource
Blinding	N/A, evaluation test sets were held out sets

Reporting for specific materials, systems and methods

We require information from authors about some types of materials, experimental systems and methods used in many studies. Here, indicate whether each material, system or method listed is relevant to your study. If you are not sure if a list item applies to your research, read the appropriate section before selecting a response.

Materials & experimental systems

n/a	Involved in the study	
☒	☐	Antibodies
☒	☐	Eukaryotic cell lines
☒	☐	Palaeontology and archaeology
☒	☐	Animals and other organisms
☐	☒	Human research participants
☒	☐	Clinical data
☒	☐	Dual use research of concern

Methods

n/a	Involved in the study	
☒	☐	ChIP-seq
☒	☐	Flow cytometry
☒	☐	MRI-based neuroimaging

Human research participants

Policy information about studies involving human research participants

Population characteristics	For TCGA, population represents the published BRCA cohort. For hospital and medical laboratory data, population was not available or defined beyond inclusion of those cases with presence of invasive carcinoma in available tissue slides.
Recruitment	No recruitment: Retrospective study of all availability cases in an overall timeframe of 1988 -2016 across datasets. Each dataset site may have different population characteristics although this data was not collected utilized as part of the study.

Ethics oversight

The study protocol was reviewed and informed consent was waived by the Naval Medical Center San Diego (NMCSD) Institutional Review Board (IRB). This IRB covered the use of de-identified cases for the data sources used in this study.

Note that full information on the approval of the study protocol must also be provided in the manuscript.
